# The Role of Alveolar Macrophages in the Improved Protection against Respiratory Syncytial Virus and Pneumococcal Superinfection Induced by the Peptidoglycan of *Lactobacillus rhamnosus* CRL1505

**DOI:** 10.3390/cells9071653

**Published:** 2020-07-09

**Authors:** Patricia Clua, Mikado Tomokiyo, Fernanda Raya Tonetti, Md. Aminul Islam, Valeria García Castillo, Guillermo Marcial, Susana Salva, Susana Alvarez, Hideki Takahashi, Shoichiro Kurata, Haruki Kitazawa, Julio Villena

**Affiliations:** 1 Laboratory of Immunobiotechnology, Reference Centre for Lactobacilli, (CERELA-CONICET), Tucuman 4000, Argentina; pclua@cerela.org.ar (P.C.); frayatonetti@gmail.com (F.R.T.); valeriagarcia@udec.cl (V.G.C.); guillemarcial@cerela.org.ar (G.M.); ssalva@cerela.org.ar (S.S.); salvarez@cerela.org.ar (S.A.); 2 Food and Feed Immunology Group, Laboratory of Animal Products Chemistry, Graduate School of Agricultural Science, Tohoku University, Sendai 980-8572, Japan; mikado0403@gmail.com (M.T.); aminul.vmed@bau.edu.bd (M.A.I.); 3 Livestock Immunology Unit, International Education and Research Center for Food and Agricultural Immunology (CFAI), Graduate School of Agricultural Science, Tohoku University, Sendai 980-8572, Japan; 4 Laboratory of Plant Pathology, Graduate School of Agricultural Science, Tohoku University, Sendai 980-8572, Japan; hideki.takahashi.d5@tohoku.ac.jp; 5 Plant Immunology Unit, International Education and Research Center for Food Agricultural Immunology, Graduate School of Agricultural Science, Tohoku University, Sendai 980-8572, Japan; 6 Laboratory of Molecular Genetics, Graduate School of Pharmaceutical Sciences, Tohoku University, Sendai 980-8572, Japan; kurata@mail.pharm.tohoku.ac.jp

**Keywords:** immunobiotics, peptidoglycan, TLR3, viral immunity, *Streptococcus pneumoniae*, respiratory syncytial virus, alveolar macrophages, *Lactobacillus rhamnosus* CRL1505

## Abstract

The nasal priming with nonviable *Lactobacillus rhamnosus* CRL1505 (NV1505) or its purified peptidoglycan (PG1505) differentially modulates the respiratory innate immune response in infant mice, improving their resistance to primary respiratory syncytial virus (RSV) infection and secondary pneumococcal pneumonia. In association with the protection against RSV-pneumococcal superinfection, it was found that NV1505 or PG1505 significantly enhance the numbers of CD11c^+^SiglecF^+^ alveolar macrophages (AMs) producing interferon (IFN)-β. In this work, we aimed to further advance in the characterization of the beneficial effects of NV1505 and PG1505 in the context of a respiratory superinfection by evaluating whether their immunomodulatory properties are dependent on AM functions. Macrophage depletion experiments and a detailed study of their production of cytokines and antiviral factors clearly demonstrated the key role of this immune cell population in the improvement of both the reduction of pathogens loads and the protection against lung tissue damage induced by the immunobiotic CRL1505 strain. Studies at basal conditions during primary RSV or *S. pneumoniae* infections, as well as during secondary pneumococcal pneumonia, brought the following five notable findings regarding the immunomodulatory effects of NV1505 and PG1505: (a) AMs play a key role in the beneficial modulation of the respiratory innate immune response and protection against RSV infection, (b) AMs are necessary for improved protection against primary and secondary pneumococcal pneumonia, (c) the generation of activated/trained AMs would be essential for the enhanced protection against respiratory pathogens, (d) other immune and nonimmune cell populations in the respiratory tract may contribute to the protection against bacterial and viral infections, and (e) the immunomodulatory properties of NV1505 and PG1505 are strain-specific. These findings significantly improve our knowledge about the immunological mechanisms involved in the modulation of respiratory immunity induced by beneficial microbes.

## 1. Introduction

Respiratory infections are of major importance because of their capacity to cause of a high degree of morbidity and mortality in high-risk populations and to rapidly spread between countries. More than four million people die yearly as a consequence of acute respiratory infections worldwide. Globally, human respiratory syncytial virus (RSV) and *Streptococcus pneumoniae* are the most important cause of fatal respiratory infections in children [1,2,3,4,5]. Generally, RSV infections are self-limiting and restricted to the upper airways. However, in susceptible individuals such as infants or the elderly, the virus may spread to the lower tract, causing more severe symptoms. Then, a viral respiratory attack may turn an immune response into pathological, resulting in tissue injury, loss of function, and even death. In addition, clinical and epidemiologic data suggest that RSV is linked to increases in the frequency [1] and severity [2] of pneumococcal diseases. In fact, it was reported that the mortality associated to respiratory viral infections is not due to the viral infection alone, but instead, secondary bacterial pneumonia complicates many severe cases in infected hosts [3,4,5].

Several mechanisms were proposed to explain the enhanced susceptibility to secondary pneumococcal pneumonia in individuals suffering primary infections with RSV. The virus-mediated impairment of the mucociliary barrier defenses and the destruction of the respiratory epithelium significantly reduce the clearance of pneumococci [6]. In addition, the upregulation of the platelet-activating factor receptor (PAF), intercellular adhesion molecule-1 (ICAM-1), and carcinoembryonic adhesion molecule 1 (CEACAM1) in respiratory epithelial cells induced by RSV increases the adherence of *S. pneumoniae* to the respiratory tract [7]. The pneumococcal adherence and colonization are also favored by the expression of the RSV G protein in respiratory epithelial surfaces that serve as an adhesion molecule for *S. pneumoniae* [6,8,9]. Moreover, it was reported that the molecular interaction of the RSV G protein with the pneumococcal penicillin-binding protein 1a induces modifications in the transcriptome of *S. pneumoniae*, increasing the expression of the virulence factors pneumolysin and neuraminidase A/B [6]. RSV has been also shown to impair respiratory immunity, adding one more mechanism by which this pathogen can increase the susceptibility to secondary infections. It was reported that RSV is capable of infecting both human and murine alveolar macrophages (AMs), although the infection is abortive, since there is no increment of viral particle production [10,11]. However, RSV induce a significant impairment in the production of interferon (IFN)-γ and interleukin (IL)-12 by AMs [12] that was associated with an enhanced severe illness in infants [13]. The reduced production of IFN-γ diminishes AM activations, impairing their phagocytic function and their ability to induce the recruitment of T and natural killer (NK) cells to the lungs, contributing to higher viral replication [14,15]. This alteration of AM functions has been associated to an exacerbated RSV-mediated bronchiolitis [15], as well as to an increased susceptibility to secondary bacterial infections [16]. Therefore, complex interactions are established between RSV, *S. pneumoniae*, and the host that need to be fully understood in order to develop strategies to diminish the severity and mortality of respiratory superinfections caused by these pathogens.

Our previous study was the first in demonstrating the ability of an immunobiotic treatment to protect against a respiratory superinfection. The nasal priming with nonviable *Lactobacillus rhamnosus* CRL1505 (NV1505) or its purified peptidoglycan (PG1505) were capable of improving the resistance of infant mice to the RSV-pneumococcal superinfection [17]. NV1505 or PG1505 differentially regulated the respiratory innate antiviral immune response triggered by the activation of Toll-like receptor 3 (TLR3), improving the resistance to a primary RSV infection and secondary pneumococcal pneumonia. The study of the immunological mechanisms involved in the protective effects induced by NV1505 and PG1505 revealed a key role for respiratory IFN-β, IFN-γ, and IL-10. Our results showed that the increase of the three cytokines in the respiratory tract induced by NV1505 or PG1505 treatments was involved in the reduction of lung RSV titers, pneumococcal colonization, bacteremia, and inflammatory-mediated lung tissue injury. Moreover, our studies provided us with preliminary information that indicated that CD11c^+^SiglecF^+^ AMs would actively participate in the beneficial effects induced by NV1505 and PG1505 by producing improved levels of IFN-β in response to pathogens [17]. However, the exact role of AMs in the immunomodulatory effects of NV1505 or PG1505, or how these respiratory immune cells are modified by the treatments to produce improved responses against RSV or pneumococcal challenges, have not been investigated in detail.

Considering this background, in this work, we aimed to further advance in the characterizations of the beneficial effects of NV1505 and PG1505 in the context of a respiratory superinfection by evaluating whether their immunomodulatory properties are dependent on AM functions. The role of AMs in the differential cytokine profiles induced in the respiratory tract by NV1505 and PG1505, as well as in their ability to increase the resistance to a primary RSV infection and secondary pneumococcal pneumonia, were explored.

## 2. Materials and Methods

### 2.1. Microorganisms and Peptidoglycans

*L. rhamnosus* CRL1505 and *L. rhamnosus* CRL498 were obtained from the CERELA culture collection (Chacabuco 145, San Miguel de Tucumán, Argentina). Both lactobacilli cultures were kept freeze-dried. Lactobacilli were cultured for 12 h at 37 °C (final log phase) in Man-Rogosa-Sharpe broth (MRS, Oxoid, Hampshire, UK). The bacteria were harvested by centrifugation at 3000× *g* for 10 min, washed three times with sterile 0.01-mol/L phosphate-buffered saline (PBS, pH 7.2), and resuspended in sterile PBS.

Nonviable *L. rhamnosus* CRL1505 (NV1505) and CRL498 (NV498) were obtained by tyndallization in a water bath at 80 °C for 30 min, and the lack of bacterial growth was confirmed using MRS agar plates. Peptidoglycan from *L. rhamnosus* CRL1505 (PG1505) and CRL498 (PG498) were obtained as described previously [17,18]. Briefly, bacteria were grown in MRS broth for 18 h at 37 °C, washed 3 times with sterile PBS, and lyophilized. Lactobacilli were resuspended in sterile water (0.1 g/mL) and lysed by sonication in an Ultrasonic Homogenizer (Cole Parmer) with cycles of 2.5 min and an amplitude of 70%. The cell wall obtained was delipidated by successive refluxing with methanol, methanol–chloroform (1:1), and chloroform. The delipidated preparation was resuspended in Tris–HCl buffer 50 μM (pH 7.5) and treated with bovine pancreatic DNAse I (Sigma Aldrich, St. Louis, MO, USA) (50 μg/mL) and ribonuclease A (Sigma Aldrich) (100 μg/mL) at 37 °C, 4 h. Finally, the cell wall was treated with 50% hydrogen chloride at 4 °C for 20 h. The peptidoglycan obtained for each individual strain was washed with sterile water, adjusted to pH 7.2, and lyophilized until use.

### 2.2. Animals and Treatments

Infant (3-week-old) BALB/c mice were obtained from the closed colony kept at CERELA (San Miguel de Tucumán, Argentina). Animals were housed in plastic cages at room temperature, and the assays for each parameter studied were performed in 5–6 mice per group for each time point. Nonviable *L. rhamnosus* strains were nasally administered to infant mice for two consecutive days at a dose of 10^8^ cells/mouse/day in 50 μL of PBS [17,19]. Peptidoglycans obtained from the different lactobacilli strains were administered individually for the nasal route to infant mice for two consecutive days at a dose of 8 μg/mL in 50 μL of PBS, as described previously [17,18]. The treated groups and the PBS-treated control group were fed a conventional balanced diet ad libitum.

This study was carried out in strict accordance with the recommendations in the Guide for the Care and Use of Laboratory Animals of the Guidelines for Animal Experimentation of CERELA. The CERELA Institutional Animal Care and Use Committee prospectively approved this research under the protocol BIOT-CRL-18. All efforts were made to minimize the number of animals and their suffering. No signs of discomfort or pain were observed before mice reached the endpoints. No deaths were observed before mice reached the endpoints.

### 2.3. Poly(I:C) Administration and Respiratory Infections

Administration of the TLR3 agonist poly(I:C) was performed two days after the last day of treatments with nonviable lactobacilli or their peptidoglycans. Mice received 100 μL of PBS containing 250-μg poly(I:C) (equivalent to 10 mg/kg body weight), which was administered dropwise via the nares [17,19,20]. Control animals received 100 μL of PBS. Mice received three doses of poly(I:C) or PBS with a 24-h rest period between each administration.

Human RSV strain A2 was grown in Vero cells, as described previously [17,19,20]. Briefly, Vero cells were grown in Dulbecco’s modified Eagle’s medium (DMEM) and infected with RSV for 3 h at 37 °C, 5% CO_2_. After infection, 7 mL of DMEM with 10% fetal bovine serum (Sigma, Tokyo, Japan), 0.1% penicillin-streptomycin (Pen/Strep) (Sigma, Tokyo, Japan), and 0.001% ciprofloxacin (Bayer, Leverkusen, Germany) was added to the flask, and cells were incubated until extensive syncytium formation was detected. Then, Vero cells were scraped, sonicated, and cell debris was removed by centrifugation at 700× *g* for 10 min at 4 °C. Virus supernatant was sucrose density gradient purified and stored in 30% sucrose at −80 °C. For in vivo infection, mice were challenged with 10^6^ plaque-forming unit (PFU) of RSV by the nasal route [17,19,20]. Viral challenge was performed two days after the last day of treatments with nonviable lactobacilli or their peptidoglycans. Lung RSV titers and tissue damage were evaluated 2 days after viral infection. The RSV immunoplaque assay was performed as described previously [17,19,21]. Primary RSV anti-F (clones 131-2A; Chemicon), anti-G (Mouse monoclonal (8C5 (9B6)) to RSV glycoprotein, Abcam, Cambridge, UK) and secondary horseradish peroxidase anti-mouse immunoglobulin (Anti-mouse IgG, HRP-linked Antibody #7076, Cell Signaling Technology, Danvers, Massachusetts, USA) antibodies were used. Individual plaques were developed using a DAB Substrate Kit (ab64238, Abcam, Cambridge, UK) following the manufacturer’s specifications. Results were expressed as log_10_ PFU/g of a lung.

*S. pneumoniae* serotype 6B was grown on blood agar for 18 h. Colonies were suspended in Todd Hewitt broth (Oxoid, Hampshire, UK), incubated overnight at 37 °C, harvested, and washed with sterile PBS. Cell density was adjusted to 4 × 10^7^ CFU/mL [17,22]. Challenge with pneumococci was performed five days after the last administration of poly(I:C) or RSV infection. Mice were sacrificed two days after *S. pneumoniae* infection. Lungs were excised, weighed, and homogenized in sterile peptone water. Homogenates were diluted appropriately, plated in duplicate on blood agar, and incubated for 18 h at 37 °C. *S. pneumoniae* was identified by standard techniques, and the results were expressed as log of CFU/g of a lung or CFU/mL of blood.

### 2.4. Lung Injury Parameters

Broncho-alveolar lavages (BAL) samples were obtained as described previously [17,22]. Briefly, the trachea was exposed and intubated with a catheter, and 2 sequential lavages were performed in each mouse by injecting sterile PBS. The recovered fluid was centrifuged for 10 min at 900× *g* and frozen at −70 °C for subsequent analyses.

Albumin content, a measure to quantitate the increased permeability of the bronchoalveolar–capillarity barrier, and lactate dehydrogenase (LDH) activity, an indicator of general cytotoxicity, were determined in the acellular BAL fluid. Albumin content was determined colorimetrically based on albumin binding to bromcresol green using an Albumin Diagnostic Kit (Wiener Lab, Buenos Aires, Argentina). LDH activity, expressed as units per liter of BAL fluid, was determined by measuring the formation of the reduced form of nicotinamide adenine dinucleotide (NAD) using the Wiener reagents and procedures (Wiener Lab).

### 2.5. In Vivo Depletion of Alveolar Macrophages

For depletion of the AMs, mice were inoculated intranasally with 50 μL of clodronate (dichloromethylene-bisphosphonate)-containing liposomes (CLP; Clophosome, Stratech, UK), as described elsewhere [11]. Mice were treated with CLP for two consecutive days. Optimal conditions of AM depletions were determined by the differential counting of BAL cells (Supplementary Figure S1). An equal treatment with empty liposomes (ELP) served as the control. BAL leukocyte counts were performed as described previously [17,19].

### 2.6. Alveolar Macrophages Primary Cultures

Primary cultures of murine AMs were performed as described elsewhere [11,23]. Macrophages were obtained from infant mice via BAL samples by using 1 mL of warm, sterile PBS containing 5-mM EDTA. AMs were transferred to new sterile tubes, washed twice in sterile PBS, and resuspended in RPMI 1640 medium with 10% FBS, 1-mM L-glutamine, and 100-U/mL penicillin-streptomycin. BAL cells were seeded in 24-well plates at a density of 10^5^ cells/well and incubated for 2 h at 37 °C in 5% CO_2_ to promote adherence. Non-adherent cells were washed, and AMs were maintained in a culture in RPMI 1640 medium with 10% FBS, 1-mM L-glutamine, and 100-U/mL penicillin-streptomycin at 37 °C in 5% CO_2_ for 24 h before stimulation. AMs were stimulated with poly(I:C) (50 ug/mL), *S. pneumoniae* (MOI of 3) or RSV (MOI of 5). Supernatants were collected before (basal conditions) and twenty-four hours after stimulations for cytokines analysis. In addition, the mRNA was extracted from AMs twelve hours after the RSV challenge for the evaluation of cytokines and antiviral factor gene expressions.

### 2.7. Cytokine Concentrations in BAL and Culture Supernatants

IFN-β (Mouse IFN-beta enzyme-linked immunosorbent assay (ELISA) Kit, sensitivity: 15.5 pg/mL), IFN-γ (Mouse IFN-gamma Quantikine ELISA Kit, sensitivity: 2 pg/mL), IL-6 (Mouse IL-6 Quantikine ELISA Kit, sensitivity: 1.8 pg/mL), IL-10 (Mouse IL-10 Quantikine ELISA Kit, sensitivity: 5.2 pg/mL), IL-12 (Mouse IL-12 p70 DuoSet ELISA, sensitivity: 1.5 pg/mL), and IL-27 (Mouse IL-27 p28/IL-30 Quantikine ELISA Kit, sensitivity: 4.7 pg/mL) concentrations in BAL and culture supernatants samples were measured with commercially available enzyme-linked immunosorbent assay (ELISA) technique kits following the manufacturer’s recommendations (R&D Systems, MN, USA). CCL2 (Mouse MCP1 ELISA Kit (ab208979), sensitivity: 0.487 pg/mL), CCL3 (Mouse MIP1a ELISA Kit (ab200017), sensitivity: 1.95 pg/mL), CXCL2 (Mouse MIP2 ELISA Kit (ab204517), sensitivity: 0.87 pg/mL), and CXCL10 (Mouse IP-10 ELISA Kit (ab214563), sensitivity: 3.4 pg/mL) were measured with commercially available ELISA technique kits following the manufacturer’s recommendations (Abcam, Cambridge, UK).

### 2.8. PCR Quantitative Expression Analysis by Real-Time PCR

Two-step real-time quantitative PCR was performed to characterize the expression of *IFN-α, IFN-β, IFN-γ, Mx1, RNAseL, OAS1, IL-1α, IL-1β, TNF-α, IL-6, IL-10*, and *IL-27* genes in cultured AMs. Total RNA was isolated from each sample using TRIzol reagent (Invitrogen). All cDNAs were synthesized using a Quantitect Reverse-Transcription (RT) Kit (Qiagen, Tokyo, Japan) according to the manufacturer’s recommendations. Real-time quantitative PCR was carried out using a 7300 Real-Time PCR System (Applied Biosystems, Warrington, UK) and the Platinum SYBR Green qPCR SuperMix uracil-DNA glycosylase (UDG) with 6-carboxyl-X-rhodamine (ROX) (Invitrogen, Carlsbad, California, USA). The primers used in this work are given in Supplementary Table S1. The PCR cycling conditions were 2 min at 50 °C, followed by 2 min at 95 °C, and then 40 cycles of 15 s at 95 °C, 30 s at 60 °C, and 30 s at 72 °C. The reaction mixtures contained 5 μL of sample cDNA and 15 μL of the master mix, which included the sense and antisense primers. The expression of β-actin was used to normalize the cDNA levels for the differences in the total cDNA levels in the samples.

### 2.9. Flow Cytometry Analysis

Single cells from lung samples were prepared as previously described [17,22]. Briefly, lungs were removed, finely minced, and incubated for 90 min with 300 U of collagenase (Yakult Honsha Co., Tokyo, Japan) in 15 mL of RPMI 1640 medium (Sigma, Tokyo, Japan). Collagenase-treated minced lungs were gently tapped into a plastic dish in order to dissociate the tissue into single cells. Erythrocytes were depleted by hypotonic lysis, and the cells were washed with RPMI medium supplemented with 10% heat-inactivated fetal calf serum. Cells were counted using Trypan Blue exclusion and then resuspended at an appropriate concentration of 5 × 10^6^ cells/mL.

Lung cell suspensions were preincubated with anti-mouse CD32/CD16 monoclonal antibody (Fc block) for 15 min at 4 °C. Cells were incubated in the antibody mixes for 30 min at 4 °C and washed with FACS buffer. Then, cells were stained with fluorochrome-conjugated antibodies against CD11c (APC), SiglecF (PE) (BD Bioscience, San Jose, CA, USA), CD45 (FITC) (eBioscience), and MHC-II (PerCP) (Thermo Fisher Scientific, Waltham, Massachusetts, USA). Cells were then acquired on a BD FACSCalibur^TM^ flow cytometer (BD Biosciences), and data were analyzed with FlowJo software (TreeStar). The total number of cells in each population was determined by multiplying the percentages of subsets within a series of marker negative or positive gates by the total cell number determined for each tissue sample [17,22].

### 2.10. Statistical Analysis

Experiments were performed in triplicate, and results were expressed as mean ± standard deviation (SD). After verification of the normal distribution of data, 2-way ANOVA was used. Tukey’s test (for pairwise comparisons of the means) was used to test for differences between the groups. Differences were considered significant at *p* < 0.05.

## 3. Results

### 3.1. Effect of AM Depletions on the Ability of Nonviable L. rhamnosus CRL1505 and Its Peptidoglycan to Modulate the Respiratory Immune Response Triggered by Poly(I:C)

One of the most used methodologies to evaluate the role of AMs in immune responses is their depletion by administering liposomes containing toxic substances such as clodronate [11,24]. Then, we used this experimental tool to evaluate the role of AMs in the immunomodulatory effects of NV1505 and PG1505. In our hands, the nasal administration of clodronate-containing liposomes (CLP) significantly reduced the number of macrophages in BAL samples for a period of six days. The number of BAL macrophages started to recover from day seven (Supplementary Figure S1). This effect was not observed in infant mice nasally treated with empty liposomes (ELP) in which BAL macrophages numbers were normal during all the assessed periods (Supplementary Figure S1). These results indicated that CLP treatment is useful for evaluating the role of AMs in the immunomodulatory effects of NV1505 and PG1505 in our experimental models, since these immune cells are decreased at the time of NV1505 or PG1505 administration, while their number return to normality when the poly(I:C), RSV, or pneumococcal challenges occur.

**Figure 1:**
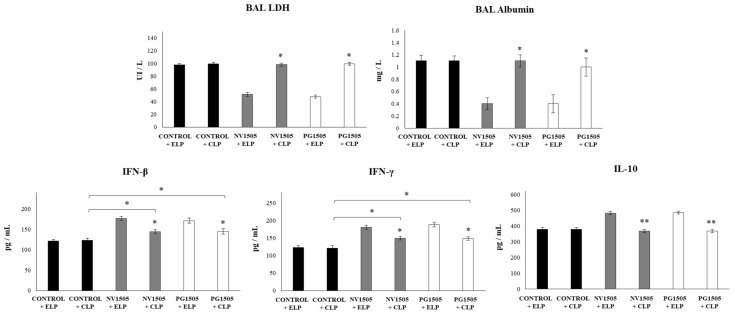
Effects of alveolar macrophage depletions on the ability of nonviable *Lactobacillus rhamnosus* CRL1505 and its peptidoglycan to modulate the respiratory immune response triggered by the poly(I:C) treatment. Infant mice were nasally treated with clodronate-containing liposomes (CLP), and four days after the last CPL administration, mice were nasally primed with nonviable *L. rhamnosus* CRL1505 (NV1505) or its peptidoglycan (PG1505) during two consecutive days and challenged with three once-daily doses of poly(I:C). Mice treated with empty liposomes (ELP) were used as controls. Two days after the last poly(I:C) administration, lactate dehydrogenase (LDH) activity, albumin concentrations, and the levels of interferon (IFN)-β, IFN-γ, and interleukin (IL)-10 in bronchoalveolar lavages (BAL) were evaluated. The results represent data from three independent experiments. Asterisks indicate significant differences between the respective ELP and CLP groups. Asterisks in black lines indicate significant differences between the indicated groups. * (*p* < 0.05) and ** (*p* < 0.01).

In accordance with the results reported previously [17], it was observed that, in infant mice treated with ELP and challenged with poly(I:C), the levels of BAL LDH and albumin increased significantly after TLR3 activation (Figure 1). It was also observed that, in animals treated with ELP and NV1505 or PG1505 and subsequently challenged with poly(I:C), the levels of BAL LDH and albumin were significantly lower than those found in their respective control group (ELP control mice) (Figure 1). The administration of CLP to control poly(I:C)-challenged mice did not induce modifications in the values of lung damage markers with respect to ELP control animals. It was also observed that, in infant mice treated with CLP and NV1505 or PG1505, the levels of BAL LDH and albumin after the challenge with poly(I:C) were significantly higher than those observed ELP+NV1505 and ELP+PG1505 mice (Figure 1). Moreover, the levels of injury markers in CLP+NV1505 and CLP+PG1505 mice were not different from the CLP control group.

The levels of IFN-β, IFN-γ, and IL-10 in BAL were also determined after the administration of poly(I:C) in infant mice treated with CLP or ELP (Figure 1). The administration of the TLR3 agonist induced significant increases in the levels of BAL IFN-β, IFN-γ, and IL-10 in the ELP control mice, which were similar to those previously reported [17]. In infant mice treated with CLP, the values of the three cytokines were not different from the ELP control group. It was also found that both NV1505 and PG1505 induced significant increases in the levels of BAL IFN-β and IFN-γ, as well as in IL-10, after the administration of poly(I:C) in ELP-treated mice that were significantly higher than that observed in ELP controls (Figure 1). Both NV1505 and PG1505 treatments significantly increased BAL IFN-β and IFN-γ levels after the administration of poly(I:C) in infant mice treated with CLP with respect to the CLP control group. However, the values of both antiviral factors in mice treated with CLP+NV1505 and CLP+PG1505 were significantly lower than those found in their respective control groups: ELP+NV1505 and ELP+PG1505 (Figure 1). In addition, it was observed that, in infant mice that received CLP, the treatments NV1505 or PG1505 were unable to modify BAL IL-10 values after the challenge with poly(I:C) when compared to the CLP control group (Figure 1).

### 3.2. Effects of AM Depletions on the Ability of Nonviable L. rhamnosus CRL1505 and Its Peptidoglycan to Modulate the Resistance to Secondary Pneumococcal Pneumonia after Poly(I:C) Treatment

**Figure 2:**
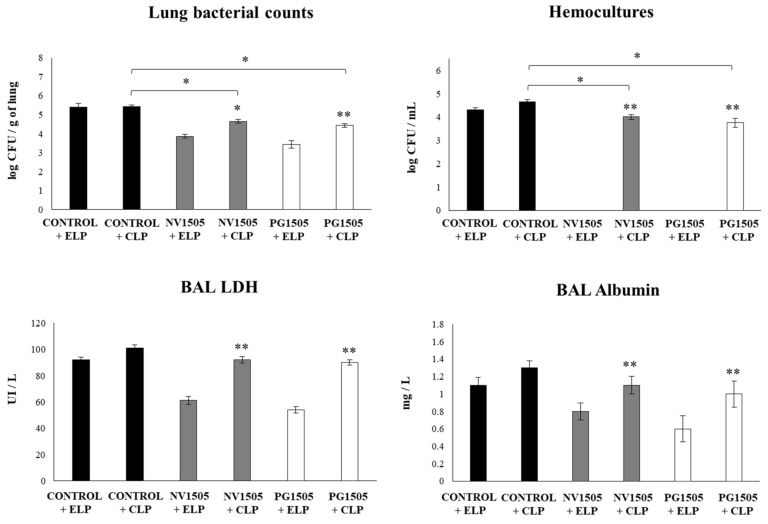
Effects of alveolar macrophage depletions on the ability of nonviable *L. rhamnosus* CRL1505 and its peptidoglycan to improve the resistance to secondary pneumococcal pneumonia after poly(I:C) treatment. Infant mice were nasally treated with clodronate-containing liposomes (CLP) during two days, and four days after the last CPL administration, mice were nasally primed with nonviable *L. rhamnosus* CRL1505 (NV1505) or its peptidoglycan (PG1505) during two consecutive days, challenged with three once-daily doses of poly(I:C), and infected with *Streptococcus pneumoniae* five days after the last poly(I:C) administration. Mice treated with empty liposomes (ELP) were used as controls. Lung bacterial cells counts, hemocultures, lactate dehydrogenase (LDH) activity, and albumin concentrations in bronchoalveolar lavages (BAL) were determined on day 2 post-pneumococcal challenge. The results represent data from three independent experiments. Asterisks indicate significant differences between the respective ELP and CLP groups. Asterisks in black lines indicate significant differences between the indicated groups. * (*p* < 0.05) and ** (*p* < 0.01).

We next evaluated whether the in vivo depletion of AMs affected the capacity of NV1505 or PG1505 to improve the resistance against secondary pneumococcal pneumonia. For this purpose, infant mice were treated with CLP or ELP, stimulated with NV1505 or PG1505, and then challenged with poly(I:C) and *S. pneumoniae*. The challenge of ELP-treated mice with *S. pneumoniae* induced a pulmonary colonization of the respiratory pathogen and its dissemination to blood, as well as increases in the biochemical markers of lung damage (Figure 2). The values of those parameters were not different to those described previously in control infant mice [17]. It was also observed that both NV1505 and PG1505 were able to significantly reduce *S. pneumoniae* counts in the lung, prevent its dissemination into blood, and reduce BAL albumin and LDH values in ELP-treated infant mice compared to ELP controls (Figure 2). On the other hand, the treatment of animals with CLP did not modify the levels of *S. pneumoniae* counts in the lungs and blood or the levels of BAL albumin and LDH when compared to infant mice in the ELP control group (Figure 2). Mice in the CLP+NV1505 and CLP+PG1505 groups had significantly higher lung and blood bacterial cell counts, as well as levels of injury markers, when compared to the ELP+NV1505 and ELP+PG1505 groups, respectively. The values of BAL albumin and LDH in CLP+NV1505 and CLP+PG1505 mice were not different from the CLP control group. However, *S. pneumoniae* counts in the lungs and blood of CLP+NV1505 and CLP+PG1505 mice were significantly lower than that found in the CLP control group (Figure 2).

**Figure 3:**
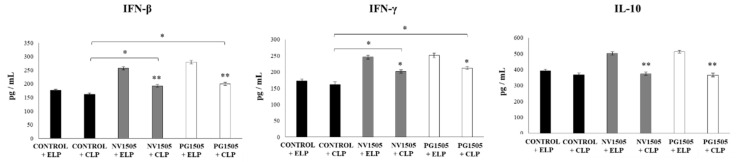
Effects of alveolar macrophage depletions on the ability of nonviable *L. rhamnosus* CRL1505 and its peptidoglycan to improve the resistance to secondary pneumococcal pneumonia after poly(I:C) treatment. Infant mice were nasally treated with clodronate-containing liposomes (CLP) during two days, and four days after the last CPL administration, mice were nasally primed with nonviable *L. rhamnosus* CRL1505 (NV1505) or its peptidoglycan (PG1505) during two consecutive days, challenged with three once-daily doses of poly(I:C), and infected with *S. pneumoniae* five days after the last poly(I:C) administration. Mice treated with empty liposomes (ELP) were used as controls. The levels of interferon (IFN)-β, IFN-γ, and interleukin (IL)-10 in bronchoalveolar lavages (BAL) were evaluated on day 2 post-pneumococcal challenge. The results represent data from three independent experiments. Asterisks indicate significant differences between the respective ELP and CLP groups. Asterisks in black lines indicate significant differences between the indicated groups. * (*p* < 0.05) and ** (*p* < 0.01).

The levels of BAL IFN-β, IFN-γ, and IL-10 were also determined after the pneumococcal infection in infant mice treated with liposomes (Figure 3). The challenge with *S. pneumoniae* induced significant increases in the levels of BAL IFN-β, IFN-γ, and IL-10 in all the experimental groups, and no differences were detected when the ELP and CLP control groups were compared (Figure 3). It was also observed that both NV1505 and PG1505 induced significant increases in the levels of BAL IFN-β, IFN-γ, and IL-10 after the pneumococcal challenge of infant mice treated with ELP. In addition, NV1505 and PG1505 significantly augmented BAL IFN-β and IFN-γ levels in infant mice treated with CLP when compared to the CLP control group. However, the values of both antiviral factors in CLP+NV1505 and CLP+PG1505 mice were significantly lower than those found in the ELP+NV1505 and ELP+PG1505 groups (Figure 3). In infant mice that received CLP, the treatments with NV1505 or PG1505 were unable to modify BAL IL-10 values after the pneumococcal challenge when compared to the CLP control group.

The effects of AM depletions in the immunomodulatory activities of NV1505 and PG1505 in the context of primary pneumococcal infections were also assessed (Supplementary Figure S2). It was observed that both NV1505 and PG1505 treatments were capable of significantly reducing *S. pneumoniae* counts in the lungs and blood, as well as BAL albumin and LDH values, in ELP-treated infant mice. Both treatments also improved the levels of BAL IFN-β, IFN-γ, and IL-10 in ELP-treated mice (Supplementary Figure S2). However, in CLP-treated mice, the beneficial effects of NV1505 and PG1505 were abolished, since most of the parameters evaluated after primary pneumococcal infections were not different from CLP-control mice. The only exception was the production of BAL IFN-γ, which was increased in NV1505- and PG1505-treated CLP mice when compared to the CLP controls but still significantly lower than their respective ELP groups (Supplementary Figure S2).

### 3.3. Effects of AM Depletions on the Ability of Nonviable L. rhamnosus CRL1505 and Its Peptidoglycan to Improve the Resistance to Secondary Pneumococcal Pneumonia after RSV Infection

**Figure 4:**
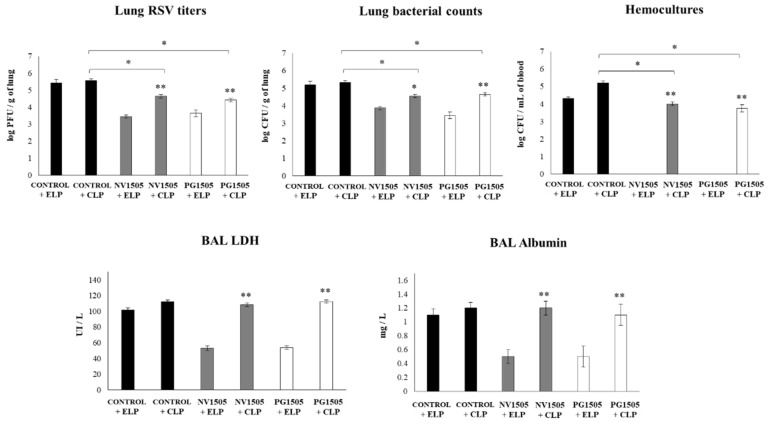
Effects of alveolar macrophage depletions on the ability of nonviable *L. rhamnosus* CRL1505 and its peptidoglycan to improve the resistance to secondary pneumococcal pneumonia after the primary infection with the respiratory syncytial virus (RSV). Infant mice were nasally treated with clodronate-containing liposomes (CLP) during two days, and four days after the last CPL administration, mice were nasally primed with nonviable *L. rhamnosus* CRL1505 (NV1505) or its peptidoglycan (PG1505) during two consecutive days, challenged with RSV, and infected with *S. pneumoniae* five days after the viral infection. Mice treated with empty liposomes (ELP) were used as controls. RSV lung titers, lung bacterial cell counts, hemocultures, lactate dehydrogenase (LDH) activity, and albumin concentrations in bronchoalveolar lavages (BAL) were determined on day 2 post-pneumococcal challenge. The results represent data from three independent experiments. Asterisks indicate significant differences between the respective ELP and CLP groups. Asterisks in black lines indicate significant differences between the indicated groups. * (*p* < 0.05) and ** (*p* < 0.01).

We further evaluated the impact of AM depletions in the ability of NV1505 and PG1505 to improve the resistance against primary infections with RSV and secondary pneumococcal pneumonia. As shown in Figure 4, infant mice treated with CLP had similar lung RSV titers than that observed in ELP-treated mice. The NV1505 and PG1505 treatments were able to reduce RSV titers in both ELP- and CLP-treated infant mice. However, the lung viral titers in CLP+NV1505 and CLP+PG1505 mice were significantly higher than those found in the ELP+NV1505 and ELP+PG1505 groups (Figure 4). It was also observed that both NV1505 and PG1505 were able to significantly reduce *S. pneumoniae* counts in the lungs, prevent its dissemination into the blood and reducing BAL albumin and LDH values in ELP-treated infant mice after the primary infection with RSV (Figure 4). In CLP-treated mice, *S. pneumoniae* counts in the lungs and blood or the levels of BAL albumin and LDH were not different from the infant mice in the ELP control group (Figure 4). Both NV1505 and PG1505 significantly reduced *S. pneumoniae* counts in the lungs and blood in CLP-treated infant mice when compared to the CLP control group. However, the values of these three parameters in CLP+NV1505 and CLP+PG1505 mice were significantly higher than that found in ELP+NV1505 and ELP+PG1505, respectively (Figure 4). The BAL LDH and albumin values had no differences when all the groups treated with CLP were compared.

**Figure 5:**
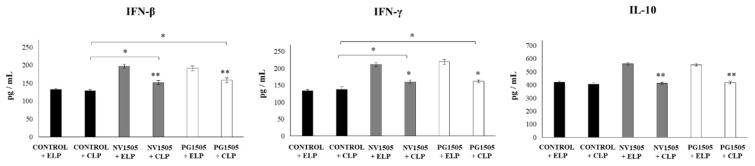
Effects of alveolar macrophage depletions on the ability of nonviable *L. rhamnosus* CRL1505 and its peptidoglycan to improve the resistance to secondary pneumococcal pneumonia after the primary infection with the respiratory syncytial virus (RSV). Infant mice were nasally treated with clodronate-containing liposomes (CLP) during two days, and four days after the last CPL administration, mice were nasally primed with nonviable *L. rhamnosus* CRL1505 (NV1505) or its peptidoglycan (PG1505) during two consecutive days, challenged with RSV, and infected with *S. pneumoniae* five days after the viral infection. Mice treated with empty liposomes (ELP) were used as controls. The levels of interferon (IFN)-β, IFN-γ, and interleukin (IL)-10 in bronchoalveolar lavages (BAL) were evaluated on day 2 post-pneumococcal challenge. The results represent data from three independent experiments. Asterisks indicate significant differences between the respective ELP and CLP groups. Asterisks in black lines indicate significant differences between the indicated groups. * (*p* < 0.05) and ** (*p* < 0.01).

The levels of BAL IFN-β, IFN-γ, and IL-10 were also determined after the secondary pneumococcal infection in infant mice treated with liposomes (Figure 5). Similar to our previous results [17], it was observed that both NV1505 and PG1505 significantly augmented the levels of BAL IFN-β, IFN-γ, and IL-10 after the pneumococcal challenge of infant mice treated with ELP. In addition, NV1505 and PG1505 significantly increased BAL IFN-β and IFN-γ levels in infant mice treated with CLP when compared to the CLP control group. However, the values of both antiviral factors in CLP+NV1505 and CLP+PG1505 mice were significantly lower than those found in the ELP+NV1505 and ELP+PG1505 groups, respectively (Figure 5). In infant mice that received CLP, the treatments with NV1505 or PG1505 were unable to modify BAL IL-10 values after the pneumococcal challenge when compared to the CLP control group.

### 3.4. Effects of Nonviable L. rhamnosus CRL1505 and Its Peptidoglycan on AMs Cytokine Profiles in Response to Different Challenges

**Figure 6:**
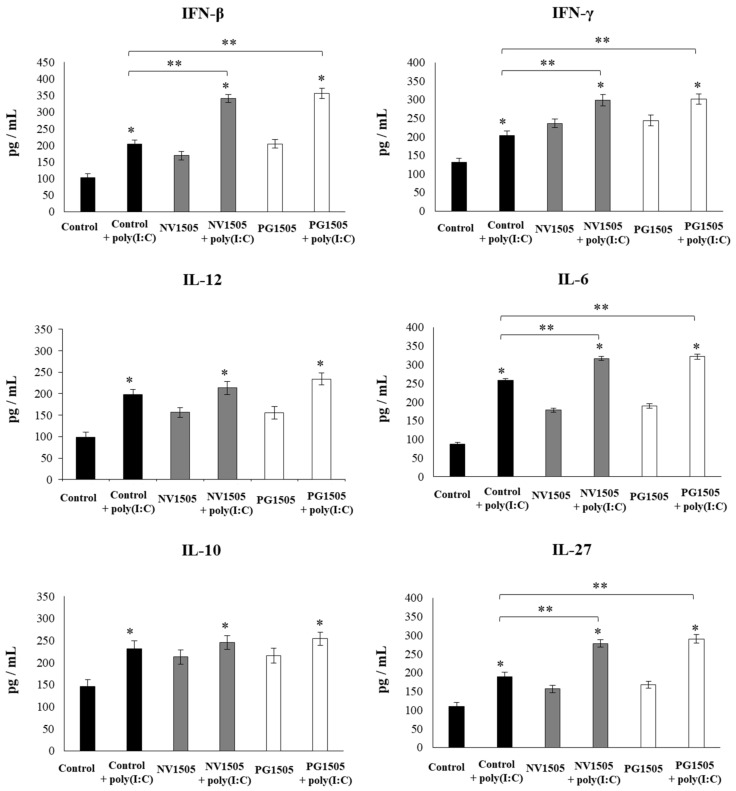
Effects of nonviable *L. rhamnosus* CRL1505 and its peptidoglycan on alveolar macrophage cytokine profiles in response to the poly(I:C) challenge. Infant mice were nasally primed with nonviable *L. rhamnosus* CRL1505 (NV1505) or its peptidoglycan (PG1505) during two consecutive days. Alveolar macrophages isolated from infant mice were challenged in vitro with poly(I:C). The levels of interferon (IFN)-β, IFN-γ, interleukin (IL)-6, IL-10, IL-12, and IL-27 were evaluated on alveolar macrophage supernatants after 24 h. The results represent data from three independent experiments. Asterisks indicate significant differences between the basal and post-poly(I:C) challenge timepoints within each group. Asterisks in black lines indicate significant differences between the indicated groups. * (*p* < 0.05) and ** (*p* < 0.01).

**Figure 7:**
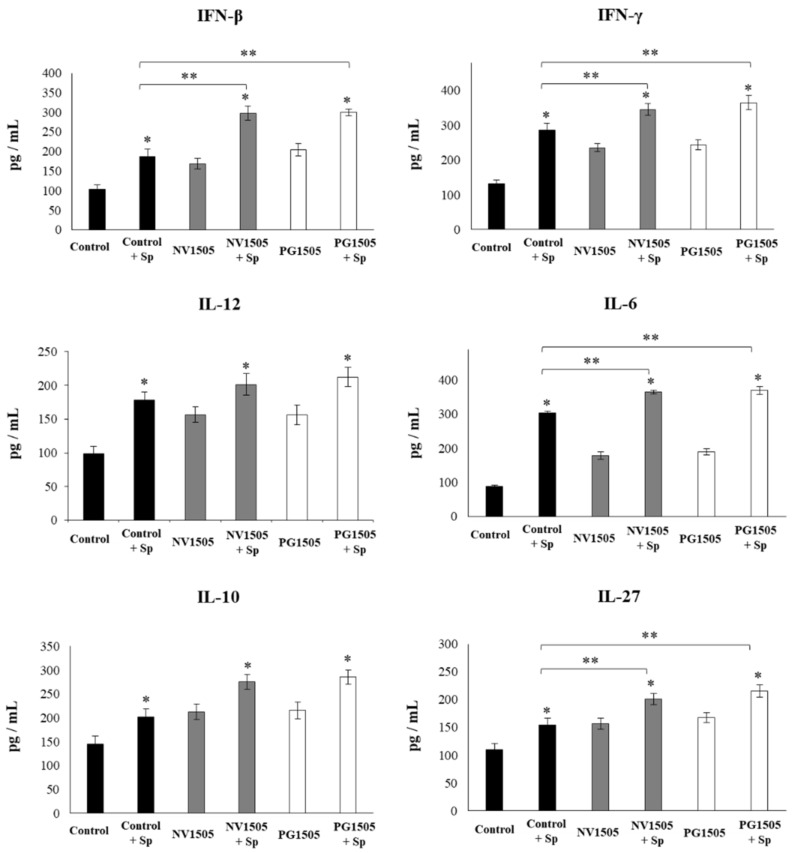
Effects of nonviable *L. rhamnosus* CRL1505 and its peptidoglycan on alveolar macrophage cytokine profiles in response to the *S. pneumoniae* challenge. Infant mice were nasally primed with nonviable *L. rhamnosus* CRL1505 (NV1505) or its peptidoglycan (PG1505) during two consecutive days. Alveolar macrophages isolated from infant mice were challenged in vitro with *S. pneumoniae*. The levels of interferon (IFN)-β, IFN-γ, interleukin (IL)-6, IL-10, IL-12, and IL-27 were evaluated on alveolar macrophage supernatants after 24 h. The results represent data from three independent experiments. Asterisks indicate significant differences between the basal and post-pneumococcal challenge timepoints within each group. Asterisks in black lines indicate significant differences between the indicated groups. * (*p* < 0.05) and ** (*p* < 0.01).

**Figure 8:**
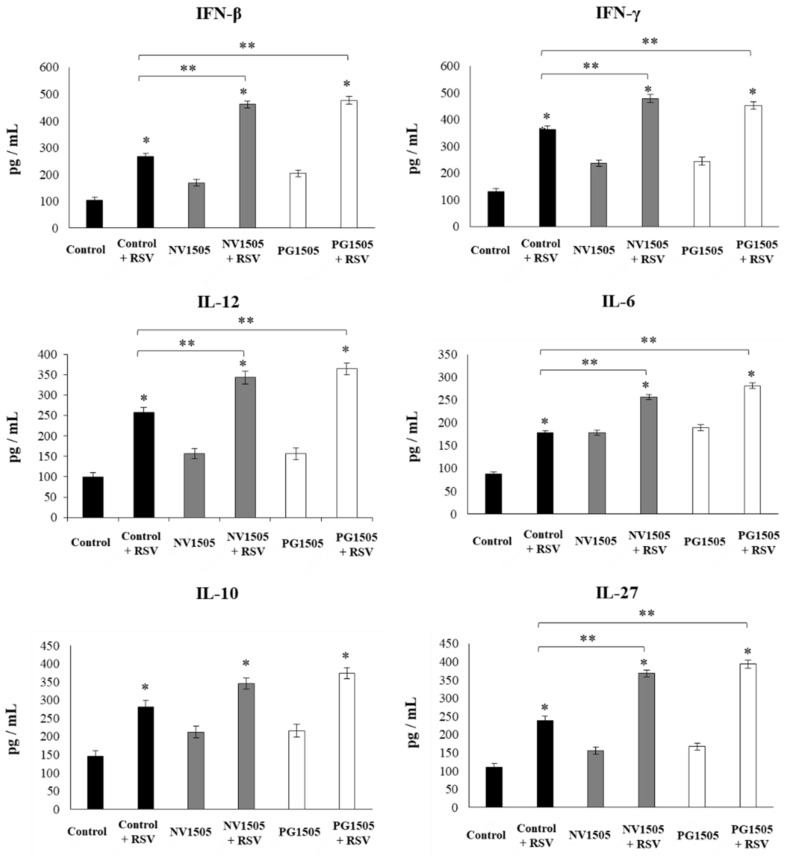
Effects of nonviable *L. rhamnosus* CRL1505 and its peptidoglycan on alveolar macrophage cytokine profiles in response to the respiratory syncytial virus (RSV) challenge. Infant mice were nasally primed with nonviable *L. rhamnosus* CRL1505 (NV1505) or its peptidoglycan (PG1505) during two consecutive days. Alveolar macrophages were isolated from infant mice and challenged in vitro with RSV. The levels of interferon (IFN)-β, IFN-γ, interleukin (IL)-6, IL-10, IL-12, and IL-27 were evaluated on alveolar macrophage supernatants after 24 h. The results represent data from three independent experiments. Asterisks indicate significant differences between the basal and post-RSV challenge timepoints within each group. Asterisks in black lines indicate significant differences between the indicated groups. * (*p* < 0.05) and ** (*p* < 0.01).

Taking into consideration that the previous results suggested that the AMs would have a relevant role in the immunomodulatory effects of NV1505 and PG1505, the changes induced by both treatments in the AM cytokine profiles in response to different stimuli were studied. For this purpose, primary cultures of AMs from the control or NV1505- or PG1505-treated infant mice were prepared, and cells were challenged in vitro with poly(I:C) (Figure 6), *S. pneumoniae* (Figure 7), or RSV (Figure 8). The basal production of IFN-β, IFN-γ, IL-6, and IL-12, as well as the immunoregulatory cytokines IL-10 and IL-27, was detected in AM cultures. Moreover, the basal levels of all the cytokines evaluated were significantly higher in AM cultures obtained from NV1505- or PG1505-treated infant mice when compared to the controls (Figure 6).

The challenges with poly(I:C) (Figure 6) or *S. pneumoniae* (Figure 7) significantly increased the levels of IFN-β, IFN-γ, IL-6, and IL-12 in the control AM cultures, as well as in those obtained from NV1505- or PG1505-treated infant mice. However, the concentrations of IL-6, IFN-β, and IFN-γ were significantly higher in AM cultures from NV1505- or PG1505-treated infant mice when compared to the control group, while IL-12 was not different from the control macrophages. In addition, both the poly(I:C) (Figure 6) and *S. pneumoniae* (Figure 7) challenges significantly increased the levels of IL-10 and IL-27 in AM cultures. However, the concentrations of IL-27 were significantly higher in AM cultures from NV1505- or PG1505-treated infant mice when compared to the control group. No differences were observed in IL-10 levels when control AMs were compared to those obtained from NV1505- or PG1505-treated infant mice (Figure 6 and Figure 7).

The challenge with RSV significantly increased IFN-β, IFN-γ, IL-6, IL-12, IL-10, and IL-27 in all the experimental groups (Figure 8). However, the concentrations of IFN-β, IFN-γ, IL-6, IL-12, and IL-27 were significantly higher in AM cultures from NV1505- or PG1505-treated infant mice when compared to the control group. No differences were observed in IL-10 levels when control AMs were compared to those obtained from NV1505- or PG1505-treated infant mice (Figure 8).

**Figure 9:**
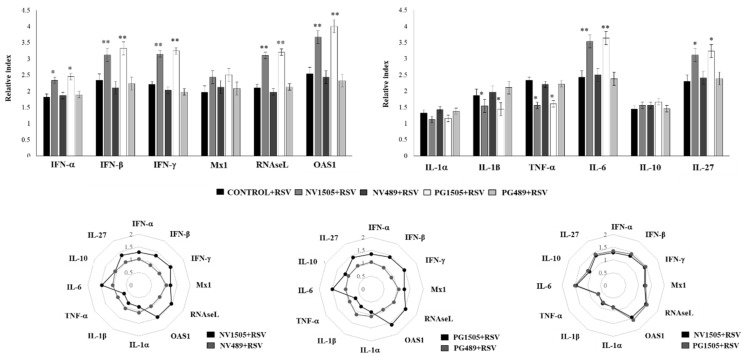
Effects of nonviable *L. rhamnosus* CRL1505 and its peptidoglycan on alveolar macrophage cytokines and antiviral factor profiles in response to the respiratory syncytial virus (RSV) challenge. Infant mice were nasally primed with nonviable *L. rhamnosus* CRL1505 or CRL489 (NV1505 or NV489) or their peptidoglycans (PG1505 or PG489) during two consecutive days. Alveolar macrophages were isolated from infant mice and challenged in vitro with RSV. The expression of *IFN-α, IFN-β, IFN-γ, Mx1, RNAseL, OAS1, IL-1α, IL-1β, TNF-1α, IL-6, IL-10*, and *IL-27* genes were evaluated in alveolar macrophages by quantitative PCR (qPCR) after 12 h. The results represent data from three independent experiments. Significant differences when compared to the control group: * (*p* < 0.05) and ** (*p* < 0.01). The multiple comparisons of the magnitude of the fold expression changes with respect to the control are shown for the NV1505 vs. NV489, PG1505 vs. PG489, and NV1505 vs. PG1505 groups.

We further characterized the response of AMs to the RSV challenge by studying the expression levels of the *IFN-α, IFN-β, IFN-γ, Mx1, RNAseL, OAS1, IL-1α, IL-1β, TNF-α, IL-6, IL-10*, and *IL-27* genes. In these experiments, animals primed with NV489 and PG489 obtained from the nonimmunomodulatory *L. rhamnosus* CRL489 strain were used for comparisons. Primary cultures from AMs obtained from the control and NV1505- and PG1505-treated mice, as well as NV489- and PG489-treated animals, were prepared and stimulated with RSV (Figure 9). As expected, the expressions of *IFN-α, IFN-β*, and *IFN-γ* in AMs from NV1505- and PG1505-treated mice were significantly higher than that observed in macrophages from the controls or mice treated with NV489 or PG489. In accordance with the improved IFN response, the levels of the antiviral factors *RNAseL* and *OAS1* in macrophages from the NV1505 and PG1505 groups were significantly higher than the control, NV489, or PG489 groups (Figure 9). Of note, the expression levels of *Mx1* were similar in all the experimental groups. The expression levels of *IL-1α* and *IL-10* were not different when the experimental groups were compared (Figure 9). The *TNF-α* and *IL-1β* expression levels were significantly lower in AMs from NV1505- and PG1505-treated mice when compared with the other groups. In addition, AMs from the NV1505 and PG1505 groups had significantly higher *IL-6* and *IL-27* expression levels when compared to the control group or those obtained from NV489- and PG489-treated animals (Figure 9). The multiple comparisons of the magnitude of the fold expression changes with respect to the control indicated clear differences between the NV1505 and NV489 groups, as well as PG1505 and PG489 (Figure 9). Moreover, both NV1505 and PG1505 were equally effective for modulating the transcriptomic response of AMs in response to the RSV challenge (Figure 9).

### 3.5. Induction of Activated/Trained AMs by Nonviable L. rhamnosus CRL1505 and Its Peptidoglycan

**Figure 10:**
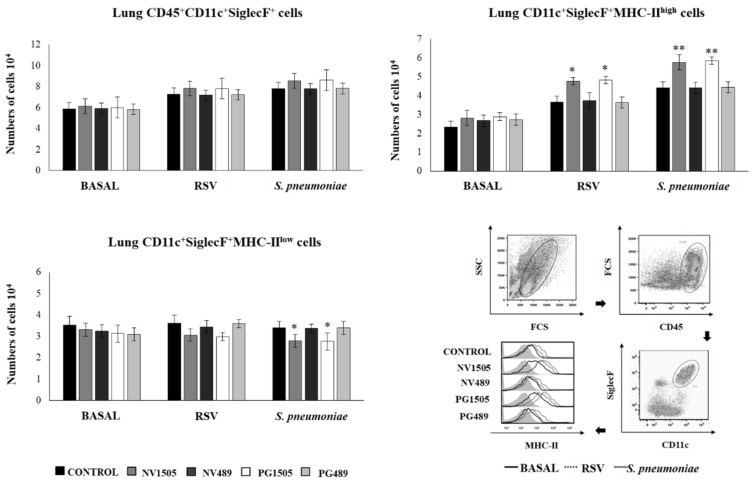
Effects of nonviable *L. rhamnosus* CRL1505 and its peptidoglycan on the alveolar macrophage numbers. Infant mice were nasally primed with nonviable *L. rhamnosus* CRL1505 or CRL489 (NV1505 or NV489) or their peptidoglycans (PG1505 or PG489) during two consecutive days, challenged with the respiratory syncytial virus (RSV), and infected with *S. pneumoniae* five days after the viral infection. Total resident alveolar macrophage populations in the lungs (CD45^+^CD11c^+^SiglecF^+^ cells), as well as their expression of MHC-II, were evaluated one day after the treatments (basal), two days after the RSV primary infection, and two days after the secondary pneumococcal challenge. Forward scatter (FSC) and side scatter (SSC). The results represent data from three independent experiments. Significant differences when compared to the control group: * (*p* < 0.05) and ** (*p* < 0.01).

It was reported that respiratory viral infections are capable of inducing a trained immunity in AMs, improving their ability to respond to a subsequent bacterial challenge [25]. These trained AMs were characterized as cells with a high MHC-II expression, as well as with a higher capacity to release cytokines and chemokines upon restimulation. Then, we next aimed to evaluate whether such kinds of trained AMs were induced during the course of the RSV-*S. pneumoniae* superinfection and the effects of NV1505 and PG1505 on this particular immune cell population. NV489 or PG489 treatments were also used for comparisons. The total resident AM populations in the lungs (CD45^+^CD11c^+^SiglecF^+^ cells) were evaluated one day after NV1505 and PG1505 treatments, two days after the primary RSV infection, or two days after the secondary pneumococcal challenge (Figure 10). The total number of AMs was not modified by the NV1505, PG1505, NV489, or PG489 treatments. The AM numbers slightly increased upon the challenges with RSV or *S. pneumoniae*, but no differences were found between the treated and control mice within the same period (Figure 10). The CD11c^+^SiglecF^+^MHC-II^high^ AM population was then evaluated. In control mice, CD11c^+^SiglecF^+^MHC-II^high^ cells represented less than 25% of the total AM population, most being AM MHC-II^low^ cells. The number of CD11c^+^SiglecF^+^MHC-II^high^ cells increased two and two point five-fold after the primary RSV infection and the secondary pneumococcal challenge, respectively (Figure 10). The treatments with NV1505 and PG1505 did not induce modifications in the number of CD11c^+^SiglecF^+^MHC-II^high^ cells at the basal time, although the mid fluorescence intensity (MFI) of the MHC-II expressions in AMs of these two groups of mice were significantly higher than the controls (Figure 10). In addition, the number of CD11c^+^SiglecF^+^MHC-II^high^ cells in NV1505- and PG1505-treated mice was higher than the controls after the primary RSV infection and the secondary pneumococcal challenge (Figure 10). Mice treated with NV489 or PG489 were not different from the controls in the three timepoints evaluated (Figure 10).

**Figure 11:**
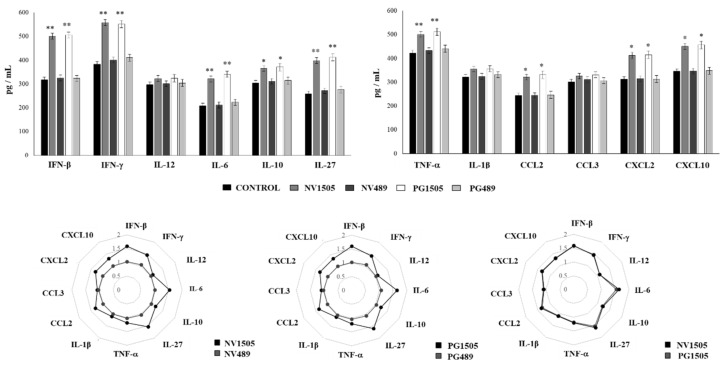
Effects of nonviable *L. rhamnosus* CRL1505 and its peptidoglycan on alveolar macrophage cytokine profiles in response to the secondary *S. pneumoniae* infection. Infant mice were nasally primed with nonviable *L. rhamnosus* CRL1505 or CRL489 (NV1505 or NV489) or their peptidoglycans (PG1505 or PG489) during two consecutive days and challenged with the respiratory syncytial virus (RSV). Alveolar macrophages isolated from infant mice were stimulated in vitro with *S. pneumoniae*. The production of IFN-β, IFN-γ, IL-6, IL-12, IL-10, IL-27, TNF-α, IL-1β, CCL2, CCL3, CXCL2, and CXCL10 was evaluated in alveolar macrophage culture supernatants after 24 h. The results represent data from three independent experiments. Significant differences when compared to the control group: * (*p* < 0.05) and ** (*p* < 0.01). The multiple comparisons of the magnitude of the fold changes with respect to the control are shown for the NV1505 vs. NV489, PG1505 vs. PG489, and NV1505 vs. PG1505 groups.

We further characterized the cytokine and chemokine profiles of AMs in response to the secondary challenge with *S. pneumoniae.* For this purpose, mice were treated with NV1505, PG1505, NV489, or PG489 and then infected with RSV. Five days after the viral challenge, AMs were collected, and primary cultures were stimulated in vitro with *S. pneumoniae*. The production of IFN-β, IFN-γ, IL-6, IL-12, IL-10, IL-27, TNF-α, IL-1β, CCL2, CCL3, CXCL2, and CXCL10 was evaluated in AM culture supernatants (Figure 11). As expected, the levels of IFN-β, IFN-γ, IL-6, IL-10, and IL-27 in AM cultures from NV1505- and PG1505-treated mice were significantly higher than that observed in AMs from the controls or mice treated with NV489 or PG489 (Figure 11). Interestingly, the production of TNF-α, CCL2, CXCL2, and CXCL10 in response to the pneumococcal challenge by AMs from the NV1505 and PG1505 groups was significantly higher than that observed in AMs from the controls and NV489- or PG489-treated mice (Figure 11). The multiple comparisons of the magnitude of the fold changes in the cytokine and chemokines productions with respect to the control indicated clear differences between the NV1505 and NV489 groups, as well as PG1505 and PG489 (Figure 11). In addition, both NV1505 and PG1505 were equally effective for modulating the cytokine/chemokine profiles of AMs in response to the secondary *S. pneumoniae* challenge (Figure 11).

**Figure 12:**
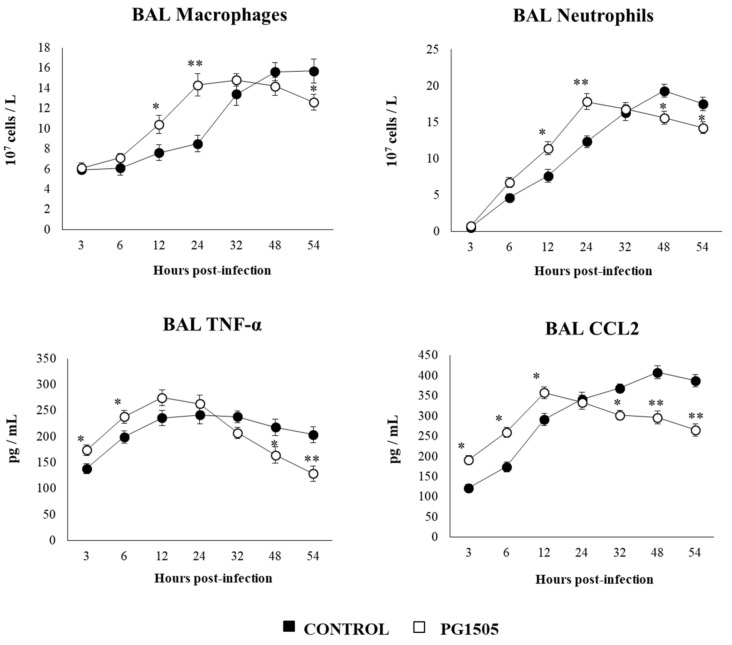
Effects of the peptidoglycan of *L. rhamnosus* CRL1505 on the immune response to the secondary *S. pneumoniae* infection. Infant mice were nasally primed with the peptidoglycan of *L. rhamnosus* CRL1505 (PG1505) during two consecutive days and challenged with the respiratory syncytial virus (RSV). Five days after the viral challenge, mice were infected with *S. pneumoniae*. The numbers of macrophages and neutrophils and the levels of TNF-α and CCL2 in bronchoalveolar lavages (BAL) were evaluated on several hours post-pneumococcal challenge. The results represent data from three independent experiments. Significant differences when compared to the control group: * (*p* < 0.05) and ** (*p* < 0.01).

Finally, we aimed to characterize in vivo the kinetics of the immune response to secondary pneumococcal pneumonia. For this purpose, mice were treated with PG1505, infected with RSV, and then challenged with *S. pneumoniae*. The levels of TNF-α and CXCL2, as well as the number of neutrophils and macrophages in the BAL samples, were determined at several points after the pneumococcal challenge (Figure 12). PG1505-treated mice had an earlier production of TNF-α and CXCL2 in the respiratory tract after the pneumococcal challenge, showing a peak at hour 12 post-infection and a trend to decrease until hour 54. Similarly, BAL neutrophil and macrophage counts in PG1505-treated mice showed a peak of cell recruitment at hour 24 post-infection and a trend to decrease until hour 54. On the contrary, control mice had a delayed increase of inflammatory cells and cytokines when compared to PG1505-treated mice. In addition, control mice had elevated levels of TNF-α and CXCL2, as well as the number of neutrophils and macrophages in the BAL samples, until the end of the studied period (Figure 12).

## 4. Discussion

AMs are the most abundant cells of the innate immune system in the lungs. They are the first immune cell population to encounter bacteria and viruses that reach the alveolar space and have a relevant role in the generation and regulation of effector responses against those pathogenic microorganisms [26]. In the present study, we focused on the specific role of AMs in the ability of nasally administered nonviable *L. rhamnosus* CRL1505 and its peptidoglycan to modulate respiratory immunity and protect against bacterial and viral pathogens. Macrophage depletion experiments and a detailed study of their production of cytokines and antiviral factors clearly demonstrated the key role of this immune cell population in the improvement of both the reduction of pathogen loads and the protection against lung tissue damage induced by the immunobiotic CRL1505 strain. Studies at basal conditions during primary RSV and *S. pneumoniae* infections and secondary pneumococcal pneumonia brought the following five notable findings regarding the immunomodulatory effects of NV1505 and PG1505: (a) AMs play a key role in the beneficial modulation of TLR3-triggered respiratory innate immune response and the protection against RSV infection, (b) AMs are necessary for the improved protection against primary and secondary pneumococcal pneumonia, (c) the generation of activated/trained AMs would be essential for the enhanced protection against respiratory pathogens, (d) other immune and nonimmune cell populations in the respiratory tract may contribute to the protection against bacterial and viral infections, and (e) the immunomodulatory properties of NV1505 and PG1505 are strain-specific.

(a) The role of AMs in the modulation of antiviral immunity by nasally administered NV1505 and PG1505. Research on the biology of RSV infection has established that AMs are required for an efficient viral clearance and control of immunopathology. The phagocytic activity of AMs is crucial for the elimination of infected cells during the course of the RSV infection. In addition, AMs play a prominent role in the defense against RSV by producing type I IFNs, which act on this same cell population or on other immune and nonimmune cells of the respiratory tract, modulating their expression of hundreds of IFN-stimulated genes (ISGs) that contribute to viral clearance [27,28]. IFN-α/β production by AMs also upregulate the expression of several chemokines in the respiratory tract that induce the recruitment of inflammatory monocytes/macrophages that further support the clearance of virus-infected cells [29]. On the other hand, it was reported that IFN-γ is of importance for the protection against the RSV [10,11]. The impairment in the production of IFN-γ by AMs was associated with an enhanced severe illness [13,14,15]. Then, the efficient and timely production of type I IFNs, ISGs, and IFN-γ by AMs is important to confer protection against the RSV. We previously reported enhanced levels of IFN-β and IFN-γ in BAL samples of infant mice treated with NV1505 or PG1505 after TLR3 activation or a RSV challenge [17,19]. In this work, we extended those previous findings by demonstrating the ability of NV1505 and PG1505 nasal treatments to enhance the production of type I IFNs and IFN-γ specifically by AMs in response to a RSV infection or poly(I:C) stimulation. Moreover, we reported here for the first time an improved expression of *OAS1* and *RNAseL* in AMs after the nasal treatments with NV1505 or PG1505. OAS1 is capable of inhibiting protein synthesis and viral growth by degrading viral and cellular RNA, and it has been shown to interfere with RSV replication [27,30]. In addition, the members of the OAS family are able to activate RNAseL. The intracellular endoribonuclease RNaseL activated by OAS molecules cleaves viral and cellular RNA, resulting in apoptosis [31]. It was reported that IFN-γ is able to upregulate the activities of OAS/RNAseL, increasing the protection against the RSV infection [32]. Then, the enhancement of these antiviral factors is consistent with the improved clearance of the RSV observed previously [17,19] and in this work. Furthermore, the identification of the differential antiviral factors and cytokines profiles induced by the CRL1505 strain in AMs indicate that the immunobiotic treatment has the potential to protect against other respiratory viruses, as we have demonstrated for the influenza virus (IFV) [33]. The precise role of AMs in the protection against the RSV was demonstrated in animal models in which this immune cell population was specifically depleted. Experiments in CD169-diphtheria toxin receptor transgenic mice, which are depleted from CD169^+^ AMs after the administration of the diphtheria toxin, demonstrated that macrophage eliminations impaired the production of IFN-β, IL-6, and TNF-α in the respiratory tract in response to the RSV infection [34]. Similar results were obtained in New Zealand black mice, which lack normal macrophage functions and show an enhanced lung immunopathology upon an RSV exposure [28]. The depletion of murine AMs by the administration of clodronate liposomes before the challenge with the RSV significantly impaired lung IFN-α, TNF-α, and IL-6 productions and diminished the activation and recruitment of NK cells. Those changes were associated to an enhanced lung RSV load [35]. By using a similar approach, we demonstrated here that the depletion of AMs by clodronate liposomes at the time of NV1505 or PG1505 nasal priming significantly diminished, but not completely abolished, the ability of treated infant mice to produce improved levels of IFN-β or IFN-γ in response to TLR3 activation or a RSV infection. These results indicate that AMs have an important role in the production of IFN-β and IFN-γ, but other respiratory cell populations may also contribute to the improved levels of both antiviral factors, as discussed below. Of note, the depletion of AMs during the nasal priming with NV1505 or PG1505 completely abolished the ability of the treatments to improve IL-10 in the respiratory tract or to reduce the biochemical markers of a lung injury after TLR3 activation. Then, AMs had an essential role in the protection induced by NV1505 and PG1505 against the lung detrimental inflammation. The infection of human AMs by the RSV stimulates the secretion of several proinflammatory cytokines, including IL-6, TNF-α, IL-1β, and IL-8 [10]. Conversely, similar experiments have described the secretion of IL-10 by these respiratory innate immune cells [10]. This ability of AMs indicates that they not only have an important role in the generation of the inflammatory response but, also, in its regulation. It was widely shown that AMs are important in the control of detrimental inflammation in the context of respiratory virus infections. The alterations of AM functions by the infection with the RSV have been associated to an exacerbated viral-mediated bronchiolitis [15]. In fact, the depletion of AMs greatly increased the recruitment of inflammatory cells to the lungs during the early stage of the RSV infection, including CD11b^hi^Gr1^hi^ neutrophils and inflammatory CD11c^hi^MHC-II^hi^CD11b^+^ dendritic cells (DCs) that contribute to a hyperresponsiveness in infected mice [11]. Notably, this deregulated inflammatory response contributes poorly to the elimination of the virus while promoting local damage and affecting the lung functions. Then, the immunoregulatory functions of AMs seems crucial for avoiding the lung inflammatory-mediated damage during the course of the RSV infection. Several mechanisms have been proposed for the immunoregulatory functions of AMs in the context of viral infections, including the phagocytosis of virus-infected apoptotic cells, preventing the release of cellular contents and the triggering of further inflammatory factor productions [36], as well as the production of IL-10 [10,36]. Interestingly, another anti-inflammatory strategy of AMs is their ability to promote Treg cell responses by directly interacting with these cells or indirectly through the production of certain cytokines [37,38]. We previously reported increased levels of IL-10 in the respiratory tracts of NV1505- or PG1505-treated mice in response to the RSV infection, although the immune cell population producing this anti-inflammatory cytokine was not determined [17,19]. The data of this work indicate that Treg cells would be the cells responsible for the improved levels of IL-10 in the respiratory tract of RSV-infected infant mice and that AMs would indirectly contribute to this effect. We demonstrated here for the first time that AMs from mice nasally primed with NV1505 or PG1505 had a significantly increased capacity to produce IL-27 in response to the RSV infection. The immunoregulatory cytokine IL-27 has been shown to protect against lung inflammatory damage during the course of viral infections. It was reported that the depletion of IL-27 enhanced the lung-damaging inflammation in RSV-infected mice [37]. In addition, it was shown that IL-27 helps in the control of the RSV infection severity by suppressing Th17- and Th2-mediated inflammations [39,40]. Interestingly, comparative studies of the IFV infections in IL-27RA^-/-^ and IL-10^-/-^ mice demonstrated that the former had a more severe disease course than the latter [41], demonstrating that not all the anti-inflammatory effects of IL-27 are mediated by the induction of IL-10 production, as it was suggested before [42,43]. Of note, it was reported that IL-27 is not sufficient for the optimal induction of Treg cell maturation in the respiratory tract and that IL-6 is required for the IL-27/Treg cell protections against inflammatory damage. The early production of IL-6 after the RSV infection induces the expression of IL-27 by myeloid cells, including AMs, which, in turn, stimulates Treg cell maturation. The depletion of IL-27 or IL-6 in the respiratory tract during the RSV have the same detrimental effect on the maturation of Treg cells and RSV-mediated immunopathology [37]. Then, our results show that the improved production of IL-27 and IL-6 by AMs of NV1505- and PG1505-treated mice may play an important role in limiting inflammation and protecting lung function during the RSV infection by increasing the maturation and activation of Treg cells. In this way, AMs indirectly increase IL-10 production in the respiratory tract of CRL1505-treated mice. It should be noted that similar results were observed when the TLR3 agonist poly(I:C) was used instead of the RSV challenge. Poly(I:C) significantly increased the production of IL-27 by AMs, which is consistent with previous findings, demonstrating that the stimulation of bone marrow-derived macrophages significantly increased the IL-27 expression in response to the TLR3 and TLR7 agonists [44]. Moreover, the ability of AMs to produce both IL-27 and IL-6 in response to TLR3 activation was enhanced in cells obtained from NV1505- and PG1505-treated mice. Then, it is tempting to speculate that NV1505 and PG1505 treatments would be capable of protecting against inflammatory-mediated lung injuries induced by other respiratory viruses with a dsRNA genome or that produce this molecule during their replication.
(b) The role of AMs in the modulation of antipneumococcal immunity by nasally administered NV1505 and PG1505. Our results also demonstrated that AMs are necessary for the improved protection against primary and secondary pneumococcal pneumonia induced by the CRL1505 strain. The depletion of AMs by clodronate liposomes at the time of NV1505 or PG1505 nasal priming abolished the ability of the treatments to reduce lung and blood pneumococcal cell counts after the primary infection while significantly diminishing, but not completely abolishing, their capacity to reduce those parameters after the secondary infection. Similar to our findings in the RSV infection experiments, improved levels of IFN-β and IFN-γ were found in the respiratory tract of NV1505- and PG1505-treated mice after the primary or secondary pneumococcal challenges. Both cytokines have been associated with the protection against this respiratory pathogen. It was demonstrated in vitro that macrophages produce type I IFNs upon *S. pneumoniae* stimulation via a mechanism dependent on bacterial uptake, and in vivo studies confirmed that AMs are the main source of type I IFNs upon the pneumococcal challenge [45]. Type I IFNs produced by AMs act on alveolar type II pneumocytes, protecting them from cell death and increasing their resistance to the *S. pneumoniae* infection [45]. Moreover, it was reported that type I IFNs increase the pulmonary barrier function and protect against the pneumococcal invasive disease [46,47,48]. The treatment with recombinant IFN-β resulted in an increased expression of tight junction proteins and the local containment of *S. pneumoniae*, while, in *ifnar1*^−/−^ mice, which have an increased susceptibility to fatal pneumococcal disease, reduced expression levels of the tight-junction genes *Tjp1*, *Cldn5*, and *Cldn18* were found [48]. On the other hand, the appropriate production of IFN-γ in the respiratory tract has been associated with the protection against *S. pneumoniae*. Improved levels of IFN-γ stimulates pulmonary macrophages that are critical for the host defenses against pneumococcal infections [49]. Genome-wide microarray-based transcriptional analysis of the whole lungs of mice infected with *S. pneumoniae* revealed that the upregulation of IFN-γ and IFN-γ-related genes was associated with the protection against this respiratory pathogen [50]. Our results show that the increase of both IFN-β and IFN-γ induced by NV1505 or PG1505 is necessary to protect against pneumococcal infections. Moreover, our data suggest that this effect may be dose-dependent. This fact would explain why AM depletions completely abolished the improved protection against the primary pneumococcal infection while only reduced the effect on the secondary infection. Other respiratory immune and nonimmune cell populations may be the sources of IFN-β and IFN-γ, which are triggered by the RSV infection and improved by NV1505 or PG1505. In addition, the depletion of AMs abolished the reduction of lung injuries and the enhancement of the respiratory IL-10 production induced by NV1505 or PG1505 in both the primary and secondary pneumococcal infections. These results are consistent with previous works reporting that the depletion of AMs before the infection with *S. pneumoniae* led to an increased local inflammatory response and enhanced mortality [51]. The improved production of IL-6 and IL-27 by AMs from NV1505- and PG1505-treated mice in response to the pneumococcal challenge suggest that these cells would also modulate the Treg cell activity and indirectly increase IL-10 production in the context of the *S. pneumoniae* infection, as it was described above for the RSV. The induction of a different cytokine profile in AMs by NV1505 or PG1505—in particular, the increases in type I IFNs, IFN-γ, IL-6, and IL-27—would be associated not only with an enhanced resistance to the primary RSV infection but, also, to the secondary pneumococcal challenge. An interesting question to answer in the future is to find out whether the immunomodulatory effects of NV1505 and PG1505 could also protect the host against the secondary bacterial pneumonia produced after the primary infection with other respiratory viruses. It should be noted that respiratory viruses are able to use different mechanisms to modulate the expressions of respiratory cytokines. In this regard, it has been shown that the increased susceptibility to the *S. pneumoniae* infection after the primary IFV challenge is associated to several changes in respiratory cytokines. The suppression of neutrophil activity and the impairment of AM functions by the excessive production of type I IFNs and IFN-γ, respectively, have been found to contribute to the enhanced susceptibility to secondary pneumococcal pneumonia [52,53,54]. In addition, it was reported that IL-27 production in an IFNAR-signaling-dependent manner induced by IFV impairs IL-17A-producing γδ T cells, leading to a reduced neutrophil response and resistance to a secondary pneumococcal infection [55]. Thus, experimental studies with other respiratory viruses are necessary to elucidate whether the immunomodulatory effects of NV1505 or PG1505 are beneficial only in post-RSV pneumococcal pneumonia or are more general. Moreover, considering that the correct regulation of both the induction and subsequent control of inflammation during respiratory infections is imperative in minimizing severe immunopathology, the detailed study of the influence of NV1505 and PG1505 on the kinetics of IFN-β, IFN-γ, IL-6, and IL-27 productions in the respiratory tract after primary viral infections and secondary pneumococcal pneumonia would be of great value to gain a better understanding of the mechanisms involved in their beneficial effects.
(c) The generation of activated/trained AMs by NV1505 and PG1505 would be essential for enhanced protection against respiratory pathogens. Recent research has revealed that, after a primary immunologic challenge, innate immune cells such as macrophages can be trained to carry a nonspecific immune memory that improves their responses to subsequent related or unrelated immunologic exposures [56,57]. Such innate immune memory has been designated as “trained immunity”, and it has been recently evaluated in the context of respiratory infections [25,57]. The induction of innate immune training in the respiratory tract was demonstrated by studies of an adenoviral infection in mice. The work reported that the respiratory viral infection induced a trained immunity phenotype in AMs characterized by an increased expression of MHC-II and release of cytokines and chemokines upon restimulation [25]. Moreover, the induction of trained AMs resulted in a higher resistance against a heterologous bacterial infection. The study also demonstrated that IFN-γ production during the primary viral infection was associated to the generation of trained AMs [25]. The role of IFN-γ in the induction of trained macrophages was supported by several subsequent studies [56,57]. Interestingly, transcriptomic studies evaluating the expression of ISGs in macrophages prestimulated it with IFN-γ in response to a second stimulation with the same cytokine demonstrated the existence of an IFN memory. Some ISGs, including *Mx1, Irf7, Ifi44, Nos2, Il12br, Ciita*, and *Tlr11*, were expressed earlier and/or in higher levels in IFN-γ-prestimulated macrophages than in naïve cells [56]. In addition, in vivo studies in which IFN-γ primed AMs were subjected to a secondary challenge revealed a rapid upregulation of IFN-γ and STAT1 signaling pathways [57]. Moreover, primed AMs had improved proinflammatory cytokine responses upon a secondary exposure to *Cryptococcus neoformans*. The results of this work show some similarities with those mentioned reports. Steady-state mouse resident AMs do not express CX3CR1 or CD11b, while they have high levels of CD11c and Siglec-F expressions (reviewed in [26]). Our studies focused on this resident CD45^+^CD11c^+^SiglecF^+^ AMs, demonstrated that the nasal priming with NV1505 or PG1505 significantly increased their expression of MHC-II two days after the RSV and *S. pneumoniae* infections, which correspond to 7 and 12 days after the CRL1505 treatments, respectively. This is consistent with studies demonstrating that trained CD11c^+^CD64^+^SiglecF^+^ AMs began to develop between 5–7 days after the primary stimuli [25]. In addition, AMs from NV1505- and PG1505-treated infant mice had an improved in vitro production of IFN-γ, TNF-α, IL-6, CCL2, CXCL2, and CXCL10, which was correlated with a more intense and faster inflammatory response in vivo upon a secondary pneumococcal challenge. Then, it is tempting to speculate that the nasal priming of NV1505 or PG1505 would be capable of activating AMs and inducing trained cells that are involved in the protection of secondary infections produced after the primary RSV infection or the TLR3 activation. However, in order to define forcefully the generation of trained AMs by NV1505 or PG1505 treatments, several complementary studies are necessary that are related to the biology of the trained immunity. Specific histone modifications in methylation patterns, as well as transcription factors binding to the promoter regions of key immune genes, have been associated with the trained immunity in macrophages [56,57]. The epigenetic reprogramming of innate immune cells during the generation of trained immunity has been also strongly associated with changes in the cellular metabolism. In this sense, an increased glycolytic metabolism has been described in trained AMs after an adenovirus infection [25]. Furthermore, protection against secondary infectious challenges have been demonstrated to last for a long period: 10 to 16 weeks after the primary stimuli [25,57]. Then, future studies are needed to evaluate the specific histone modifications and/or transcription factor bindings, as well as the metabolic changes that occur after the NV1505 or PG1505 priming of AMs that likely aid in their improved response to the in vivo challenge with the RSV or *S. pneumoniae*. In addition, a more precise investigation of the duration of the immunomodulatory effects induced by NV1505 and PG1505 in AMs would help to better understand their mechanisms of action and foresee possible protections against other respiratory pathogens.
(d) Other immune and nonimmune cell populations in the respiratory tract may contribute to the improved protections against bacterial and viral infections induced by NV1505 and PG1505. As mentioned before, the depletion of AMs did not completely abolish the ability of NV1505 or PG1505 to increase the respiratory levels of IFN-β and IFN-γ, indicating that other respiratory cell populations would contribute to this effect. Another source of improved IFN-β production could be airway and lung epithelial cells. For instance, our studies in intestinal epithelial cells demonstrated that *L. rhamnosus* CRL1505 is able to increase the expression of type I IFNs and ISGs, enhancing the protections against viral infections [58,59]. The nasal priming with NV1505 or PG1505 could have a similar effect on respiratory epithelial cells that would contribute to the protection against the RSV. In addition, the production of IFN-β by epithelial cells would be involved in the protection against pneumococcal infection through the improved production of antimicrobial factors [60] and pulmonary barrier functions [45,48]. On the other hand, an earlier production of IFN-γ in response to a respiratory viral infection has been attributed to NK cells, and several studies have demonstrated the ability of nasally administered lactobacilli to improve the activity of this immune cell population (reviewed in [61]). We have not previously evaluated the effect of nasally administered *L. rhamnosus* CRL1505 or its peptidoglycan on respiratory NK cell numbers and activities, which is an interesting topic for future near research. Another potential source of the improved levels of IFN-γ in the respiratory tract of NV1505- or PG1505-treated mice is CD4^+^ T cells. In this sense, we demonstrated previously that NV1505 is capable of increasing the number of lung CD11c^+^CD11b^low^CD103^+^ DCs [19], which have been reported to be a potent inducer of Th1 responses in the respiratory tract [62]. Consistently, the number of CD4^+^IFN-γ^+^ T cells were increased as earlier as three days after the challenge with the RSV in NV1505-treated infant mice [19].
(e) The immunomodulatory properties of NV1505 and PG1505 are strain-specific. The comparative analysis performed in this work with nonviable *L. rhamnosus* CRL1505 and CRL489 and their peptidoglycans revealed that the protection against the primary RSV infection and secondary pneumococcal pneumonia induced by CRL1505 in infant mice are strain-dependent characteristics. This is consistent with our previous reports demonstrating a strain-dependent effect of lactobacilli peptidoglycan in the context of a primary pneumococcal infection in immunocompromised malnourished mice [63]. The studies with PG1505 and the peptidoglycans from the nonimmunomodulatory strain *L. rhamnosus* CRL534 or with the immunobiotic *L. plantarum* CRL1506 showed that PG1505 has unique functional properties that cannot be extended to peptidoglycans, even from other immunomodulatory lactobacilli strains. Supporting our findings, it was shown that the nasal priming with heat-inactivated *L. reuteri* F275 significantly increased the protection of mice against the pneumonia virus of mice (PVM) infection, while its purified peptidoglycan did not confer protection [64,65]. Further comparative studies with *L. rhamnosus* CRL1505 peptidoglycan and a higher number of peptidoglycans isolated from the same species in terms of their structure, as well as their interactions with the receptors and cells of the respiratory immune system, could contribute significantly to the understanding of the molecular basis of the interaction between beneficial microorganisms and the host and its impact on respiratory infections.

In conclusion, we have provided evidence of a key role of AMs in the immunomodulatory effects of nasally administered nonviable *L. rhamnosus* CRL1505 and its peptidoglycan and in their ability to increase resistance to the primary RSV infection and secondary pneumococcal pneumonia in infant mice. These findings significantly improve our knowledge about the immunological mechanisms involved in the modulation of respiratory immunity induced by beneficial microbes.

## Supplementary Materials

The following are available online at https://www.mdpi.com/2073-4409/9/7/1653/s1, Figure S1. Alveolar macrophages depletion strategy. Figure S2. Effect of alveolar macrophages depletion on the ability of non-viable *L. rhamnosus* CRL1505 and its peptidoglycan to improve the resistance to primary pneumococcal pneumonia. Figure S3. Effect of alveolar macrophages depletion on the ability of non-viable *L. rhamnosus* CRL1505 and its peptidoglycan to modulate respiratory cytokines during primary pneumococcal pneumonia. Table S1. Primer sequences used in this study.

## References

[1] References Weinberger D.M., Givon-Lavi N., Shemer-Avni Y., Bar-Ziv J., Alonso W.J., Greenberg D., Dagan R. (2013). Influence of pneumococcal vaccines and respiratory syncytial virus on alveolar pneumonia, Israel. Emer. Infect. Dis..

[2] Cebey-Lopez M., Pardo-Seco J., Gomez-Carballa A., Martinon-Torres N., Martinon-Sanchez J.M., Justicia-Grande A., Rivero-Calle I., Pinnock E., Salas A., Fink C. (2016). Bacteremia in children hospitalized with respiratory syncytial virus infection. PLoS ONE.

[3] Bosch A.A., Biesbroek G., Trzcinski K., Sanders E.A., Bogaert D. (2013). Viral and bacterial interactions in the upper respiratory tract. PLoS Pathog..

[4] Liu L., Johnson H.L., Cousens S., Perin J., Scott S., Lawn J.E., Rudan I., Campbell H., Cibulskis R., Li M. (2012). Global, regional, and national causes of child mortality: An updated systematic analysis for 2010 with time trends since 2000. Lancet.

[5] Muscedere J., Ofner M., Kumar A., Long J., Lamontagne F., Cook D., McGeer A., Chant C., Marshall J., Jouvet P. (2013). The occurrence and impact of bacterial organisms complicating critical care illness associated with 2009 influenza A(H1N1) infection. Chest.

[6] Smith C.M., Sandrini S., Datta S., Freestone P., Shafeeq S., Radhakrishnan P., Williams G., Glenn S.M., Kuipers O.P., Hirst R.A. (2014). Respiratory syncytial virus increases the virulence of *Streptococcus pneumoniae* by binding to penicillin binding protein 1a. A new paradigm in respiratory infection. Am. J. Respir. Crit. Care Med..

[7] Avadhanula V., Rodriguez C.A., Devincenzo J.P., Wang Y., Webby R.J., Ulett G.C., Adderson E.E. (2006). Respiratory viruses augment the adhesion of bacterial pathogens to respiratory epithelium in a viral species-and cell type-dependent manner. J. Virol..

[8] Avadhanula V., Wang Y., Portner A., Adderson E. (2007). Nontypeable Haemophilus influenzae and *Streptococcus pneumoniae* bind respiratory syncytial virus glycoprotein. J. Med. Microbiol..

[9] Hament J.M., Aerts P.C., Fleer A., van Dijk H., Harmsen T., Kimpen J.L., Wolfs T.F. (2005). Direct binding of respiratory syncytial virus to pneumococci: A phenomenon that enhances both pneumococcal adherence to human epithelial cells and pneumococcal invasiveness in a murine model. Pediatr. Res..

[10] Bohmwald K., Espinoza J.A., Pulgar R.A., Jara E.L., Kalergis A.M. (2017). Functional impairment of mononuclear phagocyte system by the human respiratory syncytial virus. Front. Immunol..

[11] Kolli D., Gupta M.R., Sbrana E., Velayutham T.S., Chao H., Casola A., Garofalo R.P. (2014). Alveolar macrophages contribute to the pathogenesis of human metapneumovirus infection while protecting against respiratory syncytial virus infection. Am. J. Respir. Cell Mol. Biol..

[12] Tsutsumi H., Matsuda K., Sone S., Takeuchi R., Chiba S. (1996). Respiratory syncytial virus-induced cytokine production by neonatal macrophages. Clin. Exp. Immunol..

[13] Schultz C., Richter N., Moller J.C., Bucsky P. (2001). IFN-gamma response and IL-8 plasma levels in neonates with respiratory syncytial virus bronchiolitis. Eur. Respir. J..

[14] Eichinger K.M., Egana L., Orend J.G., Resetar E., Anderson K.B., Patel R., Empey K.M. (2015). Alveolar macrophages support interferon gamma-mediated viral clearance in RSV-infected neonatal mice. Respir. Res..

[15] Harker J.A., Yamaguchi Y., Culley F.J., Tregoning J.S., Openshaw P.J. (2014). Delayed sequelae of neonatal respiratory syncytial virus infection are dependent on cells of the innate immune system. J. Virol..

[16] Cooper G.E., Pounce Z.C., Wallington J.C., Bastidas-Legarda L.Y., Nicholas B., Chidomere C., Robinson E.C., Martin K., Tocheva A.S., Christodoulides M. (2016). Viral inhibition of bacterial phagocytosis by human macrophages: Redundant role of CD36. PLoS ONE.

[17] Clua P., Kanmani P., Zelaya H., Tada A., Kober A., Salva S., Alvarez S., Kitazawa H., Villena J. (2017). Peptidoglycan from immunobiotic lactobacillus rhamnosus improves resistance of infant mice to respiratory syncytial viral infection and secondary pneumococcal pneumonia. Front. Immunol..

[18] Kolling Y., Salva S., Villena J., Marranzino G., Alvarez S. (2015). Non-viable immunobiotic Lactobacillus rhamnosus CRL1505 and its peptidoglycan improve systemic and respiratory innate immune response during recovery of immunocompromised-malnourished mice. Int. Immunopharmacol..

[19] Tomosada Y., Chiba E., Zelaya H., Takahashi T., Tsukida K., Kitazawa H., Alvarez S., Villena J. (2013). Nasally administered Lactobacillus rhamnosus strains differentially modulate respiratory antiviral immune responses and induce protection against respiratory syncytial virus infection. BMC Immunol..

[20] Villena J., Chiba E., Tomosada Y., Salva S., Marranzino G., Kitazawa H., Alvarez S. (2012). Orally administered Lactobacillus rhamnosus modulates the respiratory immune response triggered by the viral pathogen-associated molecular pattern poly(I:C). BMC Immunol..

[21] Chiba E., Tomosada Y., Vizoso-Pinto M.G., Salva S., Takahashi T., Tsukida K., Kitazawa H., Alvarez S., Villena J. (2013). Immunobiotic *Lactobacillus* rhamnosus improves resistance of infant mice against respiratory syncytial virus infection. Int. Immunopharmacol..

[22] Kanmani P., Clua P., Vizoso-Pinto M.G., Rodriguez C., Alvarez S., Melnikov V., Takahashi H., Kitazawa H., Villena J. (2017). Respiratory commensal bacteria corynebacterium pseudodiphtheriticum improves resistance of infant mice to respiratory syncytial virus and *Streptococcus pneumoniae* superinfection. Front. Microbiol..

[23] Fernandes T.D., Cunha L.D., Ribeiro J.M., Massis L.M., Lima-Junior D.S., Newton H.J., Zamboni D.S. (2016). Murine alveolar macrophages are highly susceptible to replication of coxiella burnetii phase II in vitro. Infect. Immun..

[24] Haeberle H.A., Takizawa R., Casola A., Brasier A.R., Dieterich H.J., Van Rooijen N., Gatalica Z., Garofalo R.P. (2002). Respiratory syncytial virus-induced activation of nuclear factor-kappaB in the lung involves alveolar macrophages and toll-like receptor 4-dependent pathways. J. Infect. Dis..

[25] Yao Y., Jeyanathan M., Haddadi S., Barra N.G., Vaseghi-Shanjani M., Damjanovic D., Lai R., Afkhami S., Chen Y., Dvorkin-Gheva A. (2018). Induction of autonomous memory alveolar macrophages requires T cell help and is critical to trained immunity. Cell.

[26] Joshi N., Walter J.M., Misharin A.V. (2018). Alveolar macrophages. Cell. Immunol..

[27] Goubau D., Deddouche S., Reis e Sousa C. (2013). Cytosolic sensing of viruses. Immunity.

[28] Reed J.L., Brewah Y.A., Delaney T., Welliver T., Burwell T., Benjamin E., Kuta E., Kozhich A., McKinney L., Suzich J. (2008). Macrophage impairment underlies airway occlusion in primary respiratory syncytial virus bronchiolitis. J. Infect. Dis..

[29] Goritzka M., Makris S., Kausar F., Durant L.R., Pereira C., Kumagai Y., Culley F.J., Mack M., Akira S., Johansson C. (2015). Alveolar macrophage-derived type I interferons orchestrate innate immunity to RSV through recruitment of antiviral monocytes. J. Exp. Med..

[30] Schoggins J.W., MacDuff D.A., Imanaka N., Gainey M.D., Shrestha B., Eitson J.L., Mar K.B., Richardson R.B., Ratushny A.V., Litvak V. (2014). Pan-viral specificity of IFN-induced genes reveals new roles for cGAS in innate immunity. Nature.

[31] Ibsen M.S., Gad H.H., Thavachelvam K., Boesen T., Despres P., Hartmann R. (2014). The 2′-5′-oligoadenylate synthetase 3 enzyme potently synthesizes the 2′-5′-oligoadenylates required for RNase L activation. J. Virol..

[32] Behera A.K., Kumar M., Lockey R.F., Mohapatra S.S. (2002). Adenovirus-mediated interferon gamma gene therapy for allergic asthma: Involvement of interleukin 12 and STAT4 signaling. Hum. Gene Ther..

[33] Zelaya H., Tada A., Vizoso-Pinto M.G., Salva S., Kanmani P., Aguero G., Alvarez S., Kitazawa H., Villena J. (2015). Nasal priming with immunobiotic *Lactobacillus rhamnosus* modulates inflammation-coagulation interactions and reduces influenza virus-associated pulmonary damage. Inflamm. Res. Off. J. Eur./Histamine Res. Soc..

[34] Oh D.S., Oh J.E., Jung H.E., Lee H.K. (2017). Transient depletion of CD169(+) cells contributes to impaired early protection and effector CD8(+) T cell recruitment against mucosal respiratory syncytial virus infection. Front. Immunol..

[35] Pribul P.K., Harker J., Wang B., Wang H., Tregoning J.S., Schwarze J., Openshaw P.J. (2008). Alveolar macrophages are a major determinant of early responses to viral lung infection but do not influence subsequent disease development. J. Virol..

[36] Allard B., Panariti A., Martin J.G. (2018). Alveolar Macrophages in the resolution of inflammation, tissue repair, and tolerance to infection. Front. Immunol..

[37] Pyle C.J., Uwadiae F.I., Swieboda D.P., Harker J.A. (2017). Early IL-6 signalling promotes IL-27 dependent maturation of regulatory T cells in the lungs and resolution of viral immunopathology. PLoS Pathog..

[38] Soroosh P., Doherty T.A., Duan W., Mehta A.K., Choi H., Adams Y.F., Mikulski Z., Khorram N., Rosenthal P., Broide D.H. (2013). Lung-resident tissue macrophages generate Foxp3+ regulatory T cells and promote airway tolerance. J. Exp. Med..

[39] Hunter C.A. (2005). New IL-12-family members: IL-23 and IL-27, cytokines with divergent functions. Nat. Rev. Immunol..

[40] De Almeida Nagata D.E., Demoor T., Ptaschinski C., Ting H.A., Jang S., Reed M., Mukherjee S., Lukacs N.W. (2014). IL-27R-mediated regulation of IL-17 controls the development of respiratory syncytial virus-associated pathogenesis. Am. J. Pathol..

[41] Liu F.D., Kenngott E.E., Schroter M.F., Kuhl A., Jennrich S., Watzlawick R., Hoffmann U., Wolff T., Norley S., Scheffold A. (2014). Timed action of IL-27 protects from immunopathology while preserving defense in influenza. PLoS Pathog..

[42] Pot C., Apetoh L., Awasthi A., Kuchroo V.K. (2011). Induction of regulatory Tr1 cells and inhibition of T(H)17 cells by IL-27. Semin. Immunol..

[43] Stumhofer J.S., Silver J.S., Laurence A., Porrett P.M., Harris T.H., Turka L.A., Ernst M., Saris C.J., O’Shea J.J., Hunter C.A. (2007). Interleukins 27 and 6 induce STAT3-mediated T cell production of interleukin 10. Nat. Immunol..

[44] Stumhofer J.S., Hunter C.A. (2008). Advances in understanding the anti-inflammatory properties of IL-27. Immunol. Lett..

[45] Maier B.B., Hladik A., Lakovits K., Korosec A., Martins R., Kral J.B., Mesteri I., Strobl B., Muller M., Kalinke U. (2016). Type I interferon promotes alveolar epithelial type II cell survival during pulmonary *Streptococcus pneumoniae* infection and sterile lung injury in mice. Eur. J. Immunol..

[46] Fang R., Hara H., Sakai S., Hernandez-Cuellar E., Mitsuyama M., Kawamura I., Tsuchiya K. (2014). Type I interferon signaling regulates activation of the absent in melanoma 2 inflammasome during *Streptococcus pneumoniae* infection. Infect. Immun..

[47] Koppe U., Hogner K., Doehn J.M., Muller H.C., Witzenrath M., Gutbier B., Bauer S., Pribyl T., Hammerschmidt S., Lohmeyer J. (2012). *Streptococcus pneumoniae* stimulates a STING-and IFN regulatory factor 3-dependent type I IFN production in macrophages, which regulates RANTES production in macrophages, cocultured alveolar epithelial cells, and mouse lungs. J. Immunol..

[48] LeMessurier K.S., Hacker H., Chi L., Tuomanen E., Redecke V. (2013). Type I interferon protects against pneumococcal invasive disease by inhibiting bacterial transmigration across the lung. PLoS Pathog..

[49] Gomez J.C., Yamada M., Martin J.R., Dang H., Brickey W.J., Bergmeier W., Dinauer M.C., Doerschuk C.M. (2015). Mechanisms of interferon-gamma production by neutrophils and its function during Streptococcus pneumoniae pneumonia. Am. J. of Respir. Cell Mol. Biol..

[50] Marques J.M., Rial A., Munoz N., Pellay F.X., Van Maele L., Leger H., Camou T., Sirard J.C., Benecke A., Chabalgoity J.A. (2012). Protection against Streptococcus pneumoniae serotype 1 acute infection shows a signature of Th17-and IFN-gamma-mediated immunity. Immunobiology.

[51] Knapp S., Leemans J.C., Florquin S., Branger J., Maris N.A., Pater J., van Rooijen N., van der Poll T. (2003). Alveolar macrophages have a protective antiinflammatory role during murine pneumococcal pneumonia. Am. J. Respir. Crit. Care Med..

[52] Kudva A., Scheller E.V., Robinson K.M., Crowe C.R., Choi S.M., Slight S.R., Khader S.A., Dubin P.J., Enelow R.I., Kolls J.K. (2011). Influenza A inhibits Th17-mediated host defense against bacterial pneumonia in mice. J. Immunol..

[53] Nakamura S., Davis K.M., Weiser J.N. (2011). Synergistic stimulation of type I interferons during influenza virus coinfection promotes *Streptococcus pneumoniae* colonization in mice. J. Clinic. Investig..

[54] Sun K., Metzger D.W. (2008). Inhibition of pulmonary antibacterial defense by interferon-gamma during recovery from influenza infection. Nat. Med..

[55] Cao J., Wang D., Xu F., Gong Y., Wang H., Song Z., Li D., Zhang H., Li D., Zhang L. (2014). Activation of IL-27 signalling promotes development of postinfluenza pneumococcal pneumonia. EMBO Mol. Med..

[56] Kamada R., Yang W., Zhang Y., Patel M.C., Yang Y., Ouda R., Dey A., Wakabayashi Y., Sakaguchi K., Fujita T. (2018). Interferon stimulation creates chromatin marks and establishes transcriptional memory. Proc. Natl. Acad. Sci. USA.

[57] Leopold Wager C.M., Hole C.R., Campuzano A., Castro-Lopez N., Cai H., Caballero Van Dyke M.C., Wozniak K.L., Wang Y., Wormley F.L. (2018). IFN-gamma immune priming of macrophages in vivo induces prolonged STAT1 binding and protection against *Cryptococcus neoformans*. PLoS Pathog..

[58] Albarracin L., Garcia-Castillo V., Masumizu Y., Indo Y., Islam M.A., Suda Y., Garcia-Cancino A., Aso H., Takahashi H., Kitazawa H. (2020). Efficient selection of new immunobiotic strains with antiviral effects in local and distal mucosal sites by using porcine intestinal epitheliocytes. Front. Immunol..

[59] Albarracin L., Kobayashi H., Iida H., Sato N., Nochi T., Aso H., Salva S., Alvarez S., Kitazawa H., Villena J. (2017). Transcriptomic analysis of the innate antiviral immune response in porcine intestinal epithelial cells: Influence of immunobiotic *Lactobacilli*. Front. Immunol..

[60] MacMicking J.D. (2012). Interferon-inducible effector mechanisms in cell-autonomous immunity. Nat. Rev. Immunol..

[61] Zelaya H., Alvarez S., Kitazawa H., Villena J. (2016). Respiratory antiviral immunity and immunobiotics: Beneficial effects on inflammation-coagulation interaction during influenza virus infection. Front. Immunol..

[62] Furuhashi K., Suda T., Hasegawa H., Suzuki Y., Hashimoto D., Enomoto N., Fujisawa T., Nakamura Y., Inui N., Shibata K. (2012). Mouse lung CD103+ and CD11bhigh dendritic cells preferentially induce distinct CD4+ T-cell responses. Am. J. Respir. Cell Mol. Biol..

[63] Kolling Y., Salva S., Villena J., Alvarez S. (2018). Are the immunomodulatory properties of *Lactobacillus rhamnosus* CRL1505 peptidoglycan common for all Lactobacilli during respiratory infection in malnourished mice?. PLoS ONE.

[64] Gabryszewski S.J., Bachar O., Dyer K.D., Percopo C.M., Killoran K.E., Domachowske J.B., Rosenberg H.F. (2011). *Lactobacillus*—mediated priming of the respiratory mucosa protects against lethal pneumovirus infection. J. Immunol..

[65] Garcia-Crespo K.E., Chan C.C., Gabryszewski S.J., Percopo C.M., Rigaux P., Dyer K.D., Domachowske J.B., Rosenberg H.F. (2013). *Lactobacillus* priming of the respiratory tract: Heterologous immunity and protection against lethal pneumovirus infection. Antiviral Res..

