# Supplementary material for: The Role of Alveolar Macrophages in the Improved Protection against Respiratory Syncytial Virus and Pneumococcal Superinfection Induced by the Peptidoglycan of Lactobacillus rhamnosus CRL1505
Source: Cells. 2020 Jul 9;9(7):1653. doi: 10.3390/cells9071653 (PMC7408600; doi:10.3390/cells9071653)
Supplement: Supplementary file 1 [file cells-09-01653-s001.pdf]

cells

MDPI

Article

The Role of Alveolar Macrophages in the Improved Protection Against Respiratory Syncytial Virus and Pneumococcal Superinfection Induced by the Peptidoglycan of *Lactobacillus rhamnosus* CRL1505

Patricia Clua [1,†], Mikado Tomokiyo [2,3,†], Fernanda Raya Tonetti [1,†], Md. Aminul Islam [2,3,‡], Valeria Garcia Castillo [1,2], Guillermo Marcial [1], Susana Salva [1], Susana Alvarez [1], Hideki Takahashi [4,5], Shoichiro Kurata [6], Haruki Kitazawa [2,3,*] and Julio Villena [1,2,*]

1 Laboratory of Immunobiotechnology, Reference Centre for Lactobacilli, (CERELA-CONICET), Tucuman 4000, Argentina; pclua@cerela.org.ar (P.C.); frayatonetti@gmail.com (F.R.T.); valeriagarcia@udec.cl (V.G.C.); guillemarcial@cerela.org.ar (G.M.); ssalva@cerela.org.ar (S.S.); salvarez@cerela.org.ar (S.A.)
2 Food and Feed Immunology Group, Laboratory of Animal Products Chemistry, Graduate School of Agricultural Science, Tohoku University, Sendai 980-8572, Japan; mikado0403@gmail.com (M.T.); aminul.vmed@bau.edu.bd (M.A.I.)
3 Livestock Immunology Unit, International Education and Research Center for Food and Agricultural Immunology (CFAI), Graduate School of Agricultural Science, Tohoku University, Sendai 980-8572, Japan
4 Laboratory of Plant Pathology, Graduate School of Agricultural Science, Tohoku University, Sendai 980-8572, Japan; hideki.takahashi.d5@tohoku.ac.jp
5 Plant Immunology Unit, International Education and Research Center for Food Agricultural Immunology, Graduate School of Agricultural Science, Tohoku University, Sendai 980-8572, Japan
6 Laboratory of Molecular Genetics, Graduate School of Pharmaceutical Sciences, Tohoku University, Sendai 980-8572, Japan; kurata@mail.pharm.tohoku.ac.jp
* Correspondence: haruki.kitazawa.c7@tohoku.ac.jp (H.K.); jcvillena@cerela.org.ar (J.V.); Tel.: +81-22-757-4372 (H.K.); Tel.: +54-381-4310465 (J.V.)
† These authors contributed equally to this work.
‡ JSPS Postdoctoral research fellow.

Supplementary figures and table.

Supplementary Table S1. Primer sequences used in this study.

Gene	Sense primer (5′-3′)	Antisense primer (5′-3′)
**IFN-α**	GACTCATCTGCTGCTTGGAATGCAACCCTCC	GACTCACTCCTTCTCCTCACTCAGTCTTGCC
**IFN-β**	TCTGGAGCATCTCTTGGATGGCAA	TCCAGCTCCAAGAAAGGACGAACA
**IFN-γ**	GAAAGCCTAGAAAGTCTGAATAACT	ATCAGCAGCGACTCCTTTTCCGCTT
**Mx1**	CAGAGGTCAGCAGGACATCC	TCGCTTGCACTCTGATGACT
**OAS1**	AAAAGGAGGAGCCATGGCAGT	CTGAGCCCAAGGTCCATCAG
**RNAseL**	AAGCTTCTCAGGATCGAATGTACCAAC	GAATTCTCTGTCAAAGTGCACTGGGAC
**TNF-α**	CATCTTCTCAAAAT TCGAGTGACAA	GGGAGTAGACAAGGTACAACCC
**IL-1α**	CTCTAGAGCACCATGCTACAGAC	TGGAATCCAGGGGAAACACTG
**IL-1β**	TTGACGGACCCCAAAAGATG	AGAAGGTGCTCATGTCCTCA
**IL-6**	GTTCTCTGGGAAATCGTGGA	TGTACTCCAGGTAGCTATGG
**IL-10**	GACAATAACTGCACCCACTTC	AGCTGGTCCTTTGTTTGAAAGAAA

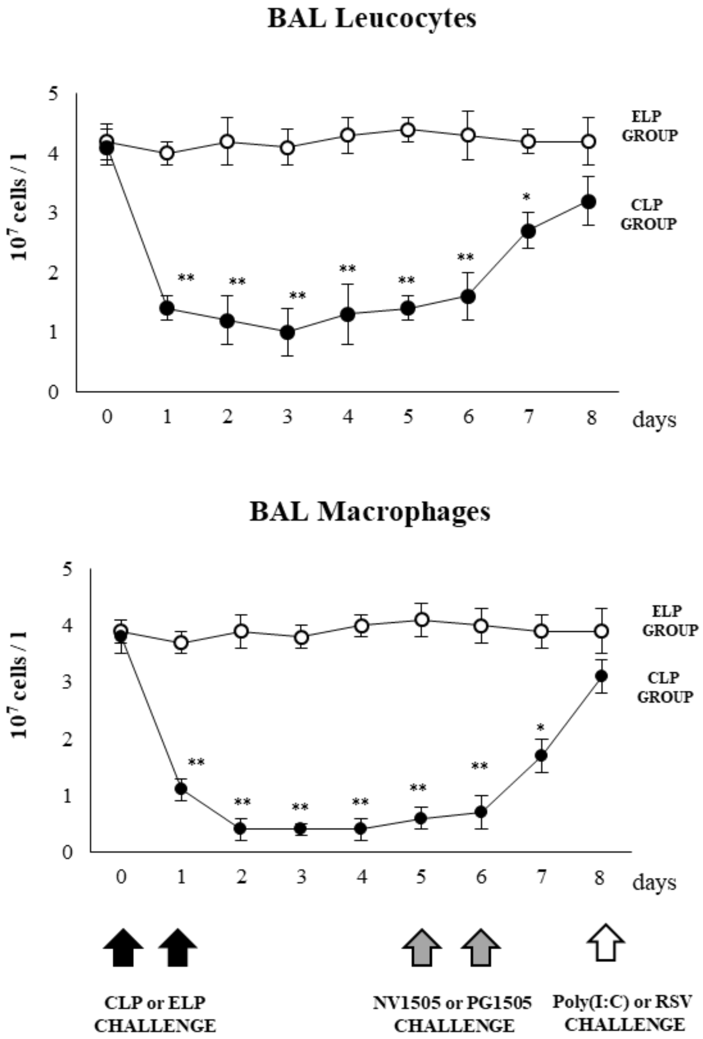

Supplementary Figure S1. Alveolar macrophages depletion strategy for the evaluation of their role on the ability of non-viable *Lactobacillus rhamnosus* CRL1505 and its peptidoglycan to modulate the respiratory immune response. Infant mice were nasally treated with clodronate-containing liposomes (CLP) during two days and four days after the last CPL administration mice were nasally primed with non-viable *L. rhamnosus* CRL1505 (NV1505) or its peptidoglycan (PG1505) during two consecutive days and challenged with three once-daily doses of poly(I:C) or infected with Respiratory Syncytial Virus (RSV). Mice treated with empty liposomes (ELP) were used as controls. The effective depletion of alveolar macrophages during the time of NV1505 or PG1505 administration was evaluated by counting total leukocytes and macrophages in bronchoalveolar lavages (BAL) samples. The results represent data from three independent experiments. Asterisks indicate significant differences between ELP and CLP groups. * ($P < 0.05$), ** ($P < 0.01$).

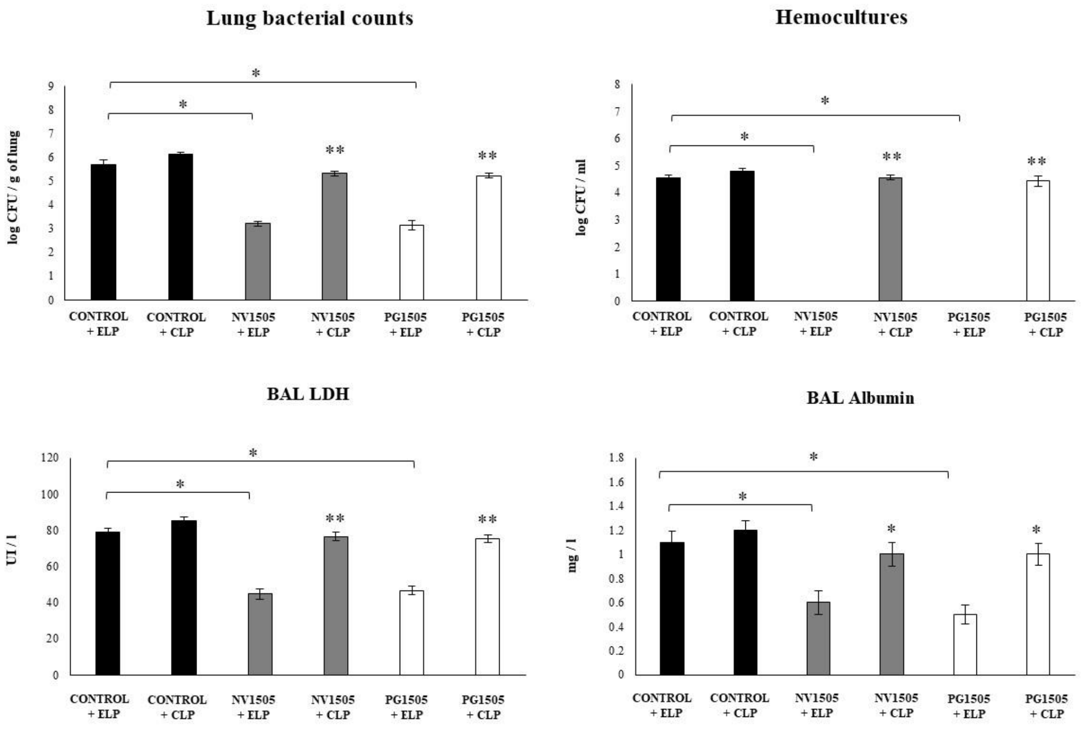

Supplementary Figure S2. Effect of alveolar macrophages depletion on the ability of non-viable *L. rhamnosus* CRL1505 and its peptidoglycan to improve the resistance to primary pneumococcal pneumonia. Infant mice were nasally treated with clodronate-containing liposomes (CLP) during two days and four days after the last CPL administration mice were nasally primed with non-viable *L. rhamnosus* CRL1505 (NV1505) or its peptidoglycan (PG1505) during two consecutive days, and infected with *S. pneumoniae*. Mice treated with empty liposomes (ELP) were used as controls. Lung bacterial cells counts, hemocultures, lactate dehydrogenase (LDH) activity and albumin concentrations in bronchoalveolar lavages (BAL) were determined on day 2 post-pneumococcal challenge. The results represent data from three independent experiments. Asterisks indicate significant differences between the respective ELP and CLP groups. Asterisks in black lines indicate significant differences between the indicated groups. * ($P < 0.05$), ** ($P < 0.01$).

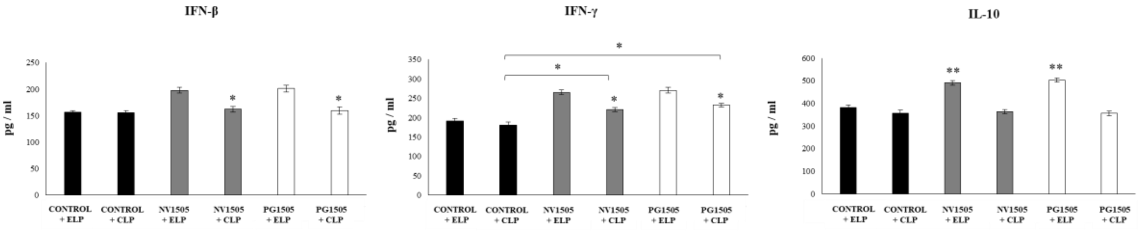

Supplementary Figure S3. Effect of alveolar macrophages depletion on the ability of non-viable *L. rhamnosus* CRL1505 and its peptidoglycan to improve the resistance to primary pneumococcal pneumonia. Infant mice were nasally treated with clodronate-containing liposomes (CLP) during two days and four days after the last CPL administration mice were nasally primed with non-viable *L. rhamnosus* CRL1505 (NV1505) or its peptidoglycan (PG1505) during two consecutive days, and infected with *S. pneumoniae*. Mice treated with empty liposomes (ELP) were used as controls. The levels of interferon (IFN)-β, IFN-γ, and interleukin (IL)-10 in bronchoalveolar lavages (BAL) were evaluated on day 2 post-pneumococcal challenge. The results represent data from three independent experiments. Asterisks indicate significant differences between the respective ELP and CLP groups. Asterisks in black lines indicate significant differences between the indicated groups. * (P < 0.05), ** (P < 0.01).
